# A TFEB–TGFβ axis systemically regulates diapause, stem cell resilience and protects against a senescence-like state

**DOI:** 10.1038/s43587-025-00911-4

**Published:** 2025-06-30

**Authors:** Tim J. Nonninger, Jennifer Mak, Birgit Gerisch, Valentina Ramponi, Kazuto Kawamura, Roberto Ripa, Klara Schilling, Christian Latza, Jonathan Kölschbach, Manuel Serrano, Adam Antebi

**Affiliations:** 1 Max Planck Institute for Biology of Ageing, Cologne, Germany; 2 Institute for Research in Biomedicine (IRB Barcelona), Barcelona Institute of Science and Technology (BIST), Barcelona, Spain; 3 Altos Labs, Cambridge Institute of Science; Granta Park, Cambridge, UK; 4 Cologne Excellence Cluster on Cellular Stress Responses in Aging-Associated Diseases (CECAD); University of Cologne, Cologne, Germany

**Keywords:** Quiescence, Senescence, Ageing

## Abstract

Diapause is a long-lived state of resilience that allows organisms to outlast adversity. *Caenorhabditis elegans* can endure months in a fasting-induced adult reproductive diapause (ARD) and, upon refeeding, regenerate and reproduce. Here we find that mutants of ARD master regulator *hlh-30/TFEB* arrest in a senescence-like state during ARD and refeeding, in which germline stem cells are characterized by DNA damage, nucleolar expansion, cell cycle arrest and mitochondrial dysfunction, alongside dysregulated immune and growth metabolic signatures, elevated senescence-associated β-galactosidase and premature aging at the organismal level. Forward genetic screens reveal a TFEB–TGFβ signaling axis that systemically controls diapause, stem cell longevity and senescence, aligning nutrient supply to proper metabolism and growth signaling. Notably, TFEB’s vital role is conserved in mouse embryonic and human cancer diapause. Thus, ARD offers a powerful model to study stem cell longevity and senescence in vivo, directly relevant to mammals.

## Abstract (web summary)

Nonninger et al. identify the TFEB–TGFβ signaling axis as a regulator of stem cell resilience that protects against a senescence-like state during the adult diapause in *Caenorhabditis elegans*, a mechanism conserved in vertebrate aging and mammalian cancer.

## Main

During food scarcity or environmental stress, organisms throughout the tree of life can persist in long-lived states of quiescence, such as diapause and torpor, that enable them to outlast adversity until conditions improve^1,2^. Diapause is an extreme form of prolonged arrest typically resistant to stress and geared toward survival, and can serve as a model of quiescence and resilience from cellular to organismal levels^3^. Indeed, its study has led to fundamental insights into aging, cellular endurance and disease. Even animals without explicit states of diapause, such as humans, globally remodel metabolism in response to fasting–refeeding, often with health benefits^4–6^. Stem cells also persist through periods of quiescence followed by activation^7^. We can thus be thought of as cellular mosaics comprising dormant diapause-like cells and actively growing or dividing non-diapause cells^4,8^, whose balance ensures stem cell longevity and organismal lifespan^4,9,10^. Notably, such mechanisms can be co-opted by tumor cells that enter diapause-like states to escape immune surveillance and resist chemotherapy^11,12^. Additionally, failure to exit cell cycle pausing may result in irreversible arrest, leading to a loss of tissue homeostasis, often described as cellular senescence^13^. Recent studies have identified only a handful of regulators of vertebrate diapause, and little is known about the mechanisms governing diapause entry, maintenance or exit and possible relationship to senescence^14^.

## Results

### HLH-30 protects against a senescence-like state, enabling dormancy and stem cell longevity

**Fig. 1:**
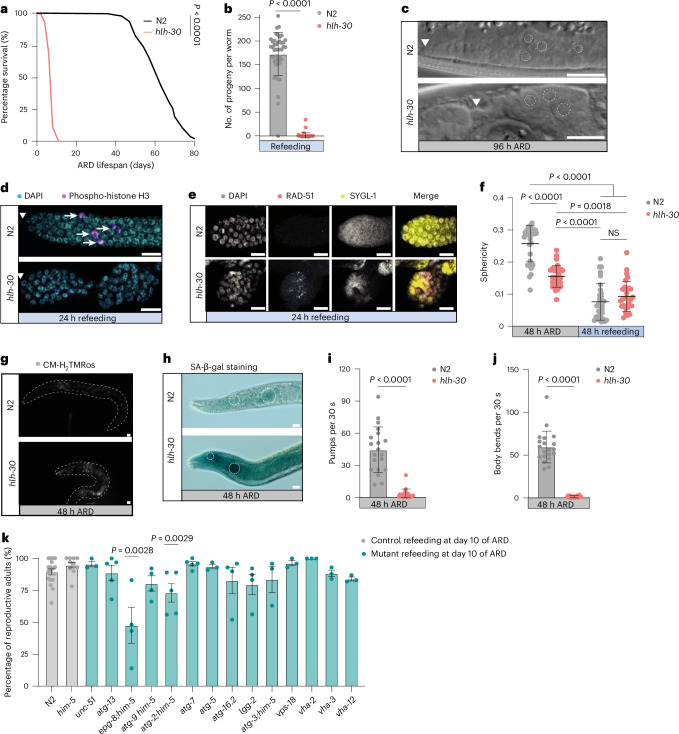
HLH-30 protects against a senescence-like state, enabling dormancy and stem cell longevity. **a**, ARD lifespan of *hlh-30(tm1978)* and N2 WT animals. **b**, Brood size of self-fertilizing worms refed after 48 h of ARD. Genotypes N2, *hlh-30(tm1978)*. Each circle represents the total progeny per worm. One representative experiment. Mann–Whitney test (two sided). Biological replicates (BR) = 3. **c**, Representative photomicrographs of germ cell nucleoli of N2, *hlh-30(tm1978)* at 96 h of ARD. Dashed circles denote germ cell nucleoli; arrowhead indicates a DTC. Scale bars, 10 μm. **d**, Distal gonad arms dissected from worms, refed at 48 h of ARD for 24 h, stained with DAPI (DNA, turquoise) and anti-phospho-histone H3 (M-phase chromosomes, magenta, arrows). Representative photomicrographs. Genotypes N2, *hlh-30(tm1978)*. Scale bars, 10 μm. Arrowhead indicates a DTC. **e**, Photomicrographs of distal gonad arms of *sygl-1::3xFlag* worms in N2 and *hlh-30(tm1978)* backgrounds, stained with DAPI (nucleus, gray), anti-RAD-51 (DNA damage foci, magenta) and anti-3xFLAG (SYGL-1, GSC zone, yellow) antibodies. Following 48 h of ARD, worms were dissected after 24 h of refeeding. Scale bars, 10 μm. **f**, Three-dimensional (3D) quantification of mitochondrial sphericity in GSCs at 48 h of ARD and 48 h of refeeding. Genotypes N2, *hlh-30(tm1978)*. Each dot represents the mitochondrial sphericity of one individual worm. Pooled data of two independent biological replicates. Two-way analysis of variance (ANOVA) followed by Tukey’s post hoc test. **g**, Representative images of mitochondrially produced ROS using CM-H_2_TMROS staining after 48 h in ARD. Scale bars, 10 μm. **h**, Representative images of SA-β-gal staining after 48 h of ARD. Dashed circles show the two pharyngeal bulbs. Scale bars, 10 μm. **i**,**j**, Pharyngeal pumping (**i**) and body bending (**j**) rates of 48-h ARD worms. 30-s intervals. Each dot represents the pumping rate or body bends of one animal. Mann–Whitney test. BR = 3, one representative experiment. **k**, Percentage reproductive worms refed after 10 days of ARD. The genotypes are shown in Table 1. Each circle represents the percentage from one biological repeat. Mann–Whitney test (two sided). Survival curves depict one experiment. All data and statistics are presented in Supplementary Table 2. If not stated otherwise, data are the mean ± s.d. NS, not significant. Source data

The nematode *Caenorhabditis elegans* is a facile model for the study of diapause. Aside from the well-characterized developmental dauer diapause^1^, the worm can enter several other quiescent states, including the ARD, a largely unstudied state of dormancy triggered in response to late larval starvation^9,15^. Such animals exhibit adult features and live over 2 months^16^. Upon refeeding, animals reactivate germline stem cells (GSCs) and regenerate somatic tissues to reproduce and live normal lifespans, revealing extraordinary survivorship and rejuvenation in the adult animal. We recently identified HLH-30/TFEB as a master regulator of ARD, whose mutation drastically reduces survivorship to 8–10 days and results in a failure to recover (Fig. 1a,b)^16^.

To better understand the role of HLH-30 during ARD and exit, we performed an in-depth phenotypic characterization of *hlh-30* mutants (Fig. 1c–j) with a focus on the adult GSCs. GSCs are the only known stem cell compartment active in the adult worm, and their activity and differentiation are regulated by the distal tip cell (DTC) niche^17^. During our studies, we observed that *hlh-30* mutants in ARD and recovery arrest in a senescence-like state. This state, not seen before in *C. elegans*, is characterized by several features of mammalian cellular senescence (Supplementary Table 1).

Mammalian cellular senescence is triggered by DNA damage, oncogene activation, mitochondrial dysfunction and other types of cellular damage^18^. Although senescent cells are heterogeneous, they are typified by enlarged cellular morphology, nucleolar expansion, cell cycle arrest, increased mitochondrial reactive oxygen species (ROS), senescence-associated β-galactosidase (SA-β-gal) activity and the senescence-associated secretory phenotype, as well as other features^19,20^.

**Extended Data Fig. 1:**
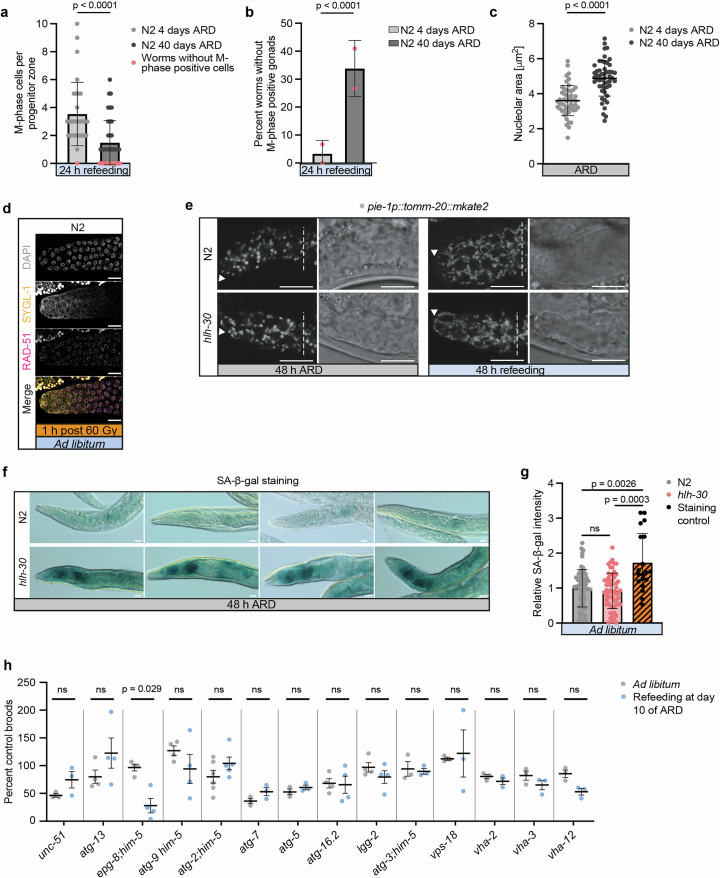
HLH-30 protects against cellular senescence, enabling dormancy and stem cell longevity. **(a)** Number of M-phase cells in the distal gonad arms (progenitor zone) of wild type (N2) recovered for 24 hours from 4 or 40 days of ARD. The salmon color indicates worms without M-phase positive cells. Pooled data from 2 independent experiments. **(b)** Percentage of N2 gonad arms negative for M-phase positive cells (salmon color) upon recovery for 24 hours from 4 or 40 days of ARD. BR = 2. **(c)** Quantification of germ cell nucleolar areas of N2 worms at 4 or 40 days of ARD. BR = 3. One representative experiment. Each dot represents one nucleolus. **(d)** Photomicrographs of distal gonad arms of *sygl-1::3xFlag* worms in N2 background, stained with DAPI (nucleus), anti-3x FLAG (SYGL-1, GSC zone, yellow) and anti-RAD-51 (DNA damage foci, magenta) antibodies. Following 1 hour post 60 Gy irradiation, ad libitum worms were dissected and stained. Scale bar 10 μm. The distal end of the gonad is facing the left side of the image. **(e)** Representative images of mKate2 labeled mitochondria in GSCs at 48 hours of ARD and 48 hours of refeeding. Images show a single z-layer of the germline, fluorescence and DIC images. Genotypes *pie-1p::tomm-20mKate* in N2 and *hlh-30(tm1978)* background. Dashed lines mark the most distal GSC area (30 μm) used for image analysis. Arrowhead show the distal end of the gonad. Scale bar 10 μm **(f)** Representative photomicrographs of head regions of 48 hour ARD worms stained with SA-β-gal. Genotypes N2, *hlh-30(tm1978)*. 4 worms per genotype. Scale bar 10 μm. **(g)** SA-β-gal RGB color intensity of head regions of young adult *hlh-30(tm1978)* worms normalized to N2. Staining control is RNAi again *bgal-2*^86^. Each dot represents the SA-β-gal intensity of one worm. Pooled data of three biological repeats. One-way ANOVA followed by Turkey’s post-hoc test. **(h)** Precent mean brood size change of autophagy and lysosomal mutants under ad libitum (grey) or refeeding after 10 days in ARD (blue). Percent change was determined in respect to N2 or *him-5* controls. Genotype alleles can be found in the reagent table. Each dots reflect one biological replicate. Kruskal-Wallis followed by Dunn’s post-hoc test. If not stated otherwise: Mean & s.d., Mann-Whitney test (two-sided). Source data

To examine GSCs in *hlh-30* and N2 wild-type (WT) animals, we induced worms into ARD for only 2–4 days, to avoid the onset of death, examining the gonads by microscopy. Additionally, we investigated *hlh-30* refeeding response by keeping worms in ARD for only 2 days, followed by 1–2 days of refeeding. While WT GSCs were compact during ARD, *hlh-30* GSCs were swollen and harbored enlarged nucleoli (Figs. 1c and 4f). Upon refeeding, WT GSCs readily entered the cell cycle (Figs. 1d and 4g), while *hlh-30* GSCs failed to do so. Importantly, irreversible cell cycle arrest and enlarged nucleoli were seen in WT animals kept long term (40 days) in ARD, suggesting WT GSCs experience similar senescence-like phenotypes with age (Extended Data Fig. 1a–c).

A common cause of cell cycle arrest is DNA damage and double-strand breaks^21^. To assess DNA damage in the GSCs, we stained the extruded gonads of animals with anti-RAD-51 antibodies and visualized foci proximal to the DTC niche by using a co-staining for the stem cell marker SYGL-1. In control WT animals maintained under ad libitum conditions and exposed to ionizing radiation, DNA damage foci were readily apparent in GSCs (Extended Data Fig. 1d). In contrast, no such foci were visible in WT experiencing ARD recovery without radiation (Figs. 1e and 4h). Interestingly, in *hlh-30* mutants, we observed an elevated fraction of animals harboring GSCs with RAD-51 foci during ARD recovery, suggesting that *hlh-30* mutants experience more DNA damage or have less efficient DNA repair.

Under physiological conditions, mitochondria dynamically switch between fusion and fission according to cellular metabolic demand^22^. With cellular senescence or aging, mitochondria can lose their dynamic flexibility and often appear fused, leading to increased ROS species^23^. We, therefore, examined mitochondrial morphology in GSCs during ARD and refeeding and ROS production during ARD. Whereas WT worm GSCs went from fragmented mitochondria (higher sphericity) during ARD to fused (lower sphericity) upon refeeding (Fig. 1f and Extended Data Fig. 1e), *hlh-30* mutant GSCs exhibited more fused mitochondria during ARD compared to WT (Fig. 1f). Upon refeeding, fusion was induced but to a lesser extent than WT. In line with this, we observed that *hlh-30* mutants showed elevated levels of oxidative stress as measured by MitoTracker CM-H_2_TMRos (Fig. 1g and Extended Data Fig. 4f), suggesting mitochondrial dysfunction similar to senescent cells in culture^24^.

On an organismal level, we found increased SA-β-gal activity in *hlh-30* mutants in ARD (Fig. 1h and Extended Data Fig. 1f), a hallmark of cellular senescence reflecting lysosomal dysfunction^20^. Importantly, we did not see changes in SA-β-gal expression under ad libitum conditions (Extended Data Fig. 1g). Alongside this, we observed a number of phenotypes consistent with organismal aging and functional decline, including decreased pharyngeal pumping activity and muscle motility (Fig. 1i,j), as well as acceleration of transcriptomic age (Fig. 4i) as measured by the BiT age algorithm^25^.

TFEB is considered a major regulator of autophagy, and dysregulation of this process accelerates cellular senescence^13^. To understand the potential role of autophagy during ARD, we examined 14 representative mutants of the autophagy cycle and lysosomal ATPase function. Mutants were kept in ARD for 10 days, refed, and the fraction of recovered reproductive animals and brood size measured. Generally, we found that most mutations (12/14) had no significant effect on the percentage of reproductive animals compared to WT, except for *epg-8/ATG-14L* and *atg-2/ATG-2A* mutations, which decreased this value (Fig. 1k). Additionally, the percentage change in brood size compared to ad libitum controls was minimal, except for *epg-8/ATG-14L*, whose mutation significantly decreased brood size (Extended Data Fig. 1h). Conceivably, this exceptional behavior is due to noncanonical roles of EPG-8/ATG-14L. We conclude that canonical autophagy is not essential for recovery during the time frame in which HLH-30 is required. Hence, other HLH-30/TFEB target processes must contribute to ARD stem cell longevity and recovery.

### Selection against ARD senescence-like state reveals longevity and stem cell resilience pathways

**Fig. 2:**
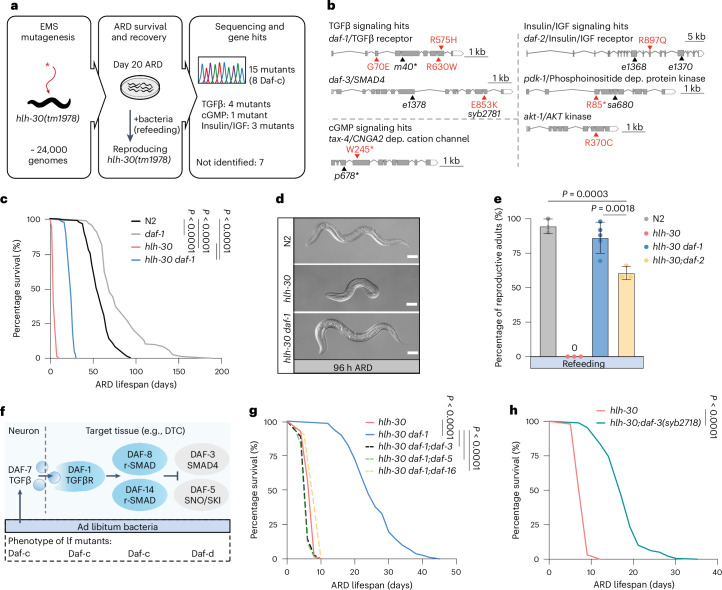
Selection against ARD senescence-like state reveals longevity and stem cell resilience pathways. **a**, Genetic screen for mutations that rescue *hlh-30(tm1978)* ARD survival and recovery. The red asterisk indicates a mutation induced by EMS. **b**, Gene structure of hits from TGFβ (*daf-1*/TGFβ receptor, *daf-3*/SMAD4), cGMP (*tax-4*/CNGA2) and insulin/IGF signaling (*daf-2*/Insulin/IGF receptor, *pdk-1*/PDPK1 and *akt-1*/AKT). Schematic intron–exon map based on WormBase gene structures. Positions of point mutation identified in this screen are marked by red arrowheads. Amino acid alterations are given in single-letter code. Reference alleles used to validate the gene hits are in black. **c**, TGFβ receptor mutation *daf-1(m40)* prolongs ARD survival of *hlh-30(tm1978)*. **d**, Representative images of worms at 96 h of ARD. Genotypes N2, *hlh-30(tm1978), hlh-30 daf-1(m40)*. Scale bars, 10 μm. **e**, Percentage of reproductive worms refed after 10 days in ARD. Genotypes N2, *hlh-30(tm1978)*, *hlh-30 daf-1(m40)* and *hlh-30;daf-2(e1370)*. Each dot represents one experiment. One-way ANOVA followed by Tukey’s post hoc test. **f**, Simplified TGFβ signaling schematic. Under ad libitum conditions, neuronal DAF-7/TGFβ induces a DAF-1/TGFβ receptor signaling cascade inhibiting repressor activity of DAF-3/SMAD4–DAF-5/SNO-SKI complex in DTC and other target tissues. Under diapause conditions, TGFβ signaling is low, and the DAF-3–DAF-5 repressor complex is activated. Below, dauer phenotypes at 25 °C of loss-of-function (lf) mutants; Daf-c, dauer formation constitutive; Daf-d, dauer formation defective^1^. **g**, *hlh-30 daf-1* ARD lifespan extension depends on *daf-3*, *daf-5* and *daf-16* encoded transcription factors. Genotypes *hlh-30(tm1978)*, *hlh-30 daf-1(m40)*, *hlh-30 daf-1;daf-3(e1376)*, *hlh-30 daf-1;daf-5(e1386)* and *hlh-30 daf-1;daf-16(mgDf50)* are all loss of function. **h**, *daf-3(syb2718)* gain-of-function mutation extends *hlh-30* ARD survival. Survival curves depict one representative experiment. Data and statistics are presented in Supplementary Table 2. If not stated otherwise, data are the mean ± s.d. Source data

To identify epistatic mechanisms acting downstream of *hlh-30*, we carried out ethyl methanesulfonate (EMS) mutagenesis of *hlh-30(tm1978)* mutants and screened for F2 suppressors that live at least 20 days, and which, upon refeeding, recover and reproduce (Fig. 2a). Whole-genome sequencing revealed that eight mutants harbored lesions in transforming growth factor beta (TGFβ) signaling (*daf-1*/TGFβR, *daf-3*/SMAD4), insulin–insulin-like growth factor (IGF) signaling (*pdk-1*/PDPK1, *akt-1*/AKT kinase, *daf-2*/Insulin/IGF receptor) and cGMP signaling (*tax-4*/CNGA1/CNGA2; Fig. 2b), the latter regulating both TGFβ and insulin-like peptide production in ciliated neurons^1,26^. Mutations in these genes are known to affect the third larval stage dauer diapause^1^ (that is, Daf mutants), suggesting an overlap of mechanisms with ARD. The remaining seven mutants were not identified because they did not map cleanly to a unique locus.

**Extended Data Fig. 2:**
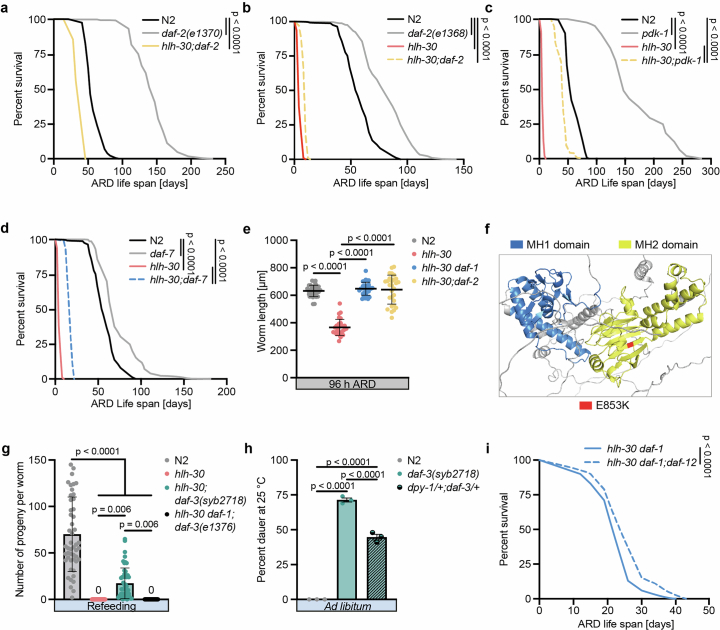
Selection against ARD senescence reveals ARD longevity and stem cell resilience pathways. **(a), (b), (c)** Insulin/IGF signaling mutants *daf-2(e1370)*, *daf-2(e1368)* and *pdk-1(sa680)* extend ARD lifespan and partially rescue *hlh-30(tm1978)* ARD short survivorship. **(d)** TGFβ signaling mutant *daf-7(e1372)* extends ARD survival and partially rescues *hlh-30(tm1978)* ARD short survivorship. **(e)** Body length of ARD worms at 96 hours. Genotypes N2, *hlh-30(tm1978), hlh-30 daf-1(m40)*, and *hlh-30;daf-2(e1370)*. Each dot represents one animal. BR = 3, one representative experiment. One-way ANOVA followed by Turkey’s post-hoc test. **(f)** Predicted protein structure (Alphafold^87^) of *daf-3(syb2718)* showing mutation E853K in the MH2 domain in red. Conserved Mad homology 1 and 2 domains (MH1 and MH2) in blue and yellow, respectively. **(g)** *daf-3(syb2718)* gain-of-function mutation restores progeny production of *hlh-30* worms refed at days 10 ARD. *daf-3(e1376)* null mutant abolishes progeny production of *hlh-30(tm1978) daf-1(m40)* double mutants. Each dot represents the progeny number of one worm. One representative replicate. BR = 3. One-way ANOVA followed by Turkey’s post-hoc test. **(h)** *daf-3(syb2718)* exhibits a dominant gain-of-function dauer constitutive (Daf-c) phenotype at 25 °C. Genotypes N2, *daf-3(syb2718)*, and *daf-3(syb2718)/+* heterozygous. Each dot represents one experiment. One-way ANOVA followed by Turkey’s post-hoc test. **(i)** *daf-12(rh61rh411)* slightly enhances ARD survival of *hlh-30(tm1978) daf-1(m40)*. Survival curves depict one representative experiment. Lifespan data and statistics (log-rank tests) are presented in Supplementary Table 2. If not stated otherwise: Mean & s.d. Source data

Identified candidate genes were validated for *hlh-30* ARD rescue by using independent reference alleles (*daf-1(m40)*, *pdk-1(sa680)*, *tax-4(p678)*, *daf-2(e1368* and *e1370*); Fig. 2c, Extended Data Fig. 2a–c and Supplementary Table 2). We also tested the *daf-7(e1372*)/TGFβ mutant and saw similar rescue (Extended Data Fig. 2d). Notably, relative fold increase in mean lifespan of *hlh-30 daf-1* double mutants compared to *hlh-30* was usually greater (>3.2–4.1-fold) than that of *daf-1* single mutants compared to WT (1.1–1.2-fold; Fig. 2c and Supplementary Table 2), suggesting a more specific interaction of TGFβ signaling with *hlh-30* within ARD. These mutations also rescued other *hlh-30*-associated traits, namely ARD body size (Fig. 2d and Extended Data Fig. 2e) and reproductive ARD recovery (Fig. 2e). Given the robust rescue of reproductive capacity as an indirect readout for GSC health, we focused mainly on TGFβ signaling for further study.

To unravel how TGFβ signaling impacts *hlh-30* ARD collapse, we first examined genetic epistasis interactions. Under ad libitum-fed conditions, DAF-7/TGFβ ligand is secreted mainly from the ASI sensory neurons and acts through TGFβ type I and II receptors, DAF-1 and DAF-4, throughout the body. This in turn activates the positive arm DAF-8 and DAF-14/r-SMADs, which inhibit the negative arm DAF-3/SMAD4 and DAF-5/SNO-SKI to promote the growth state (Fig. 2f). Under fasted conditions, TGFβ signaling is low, and DAF-3/DAF-5 become derepressed to promote the quiescent state^27^. As expected, we observed a clear genetic dependence of *daf-1*-induced lifespan rescue of *hlh-30* on downstream transcription factors *daf-3/*SMAD4, *daf-5*/SNO-SKI and *daf-16*/FOXO, another mediator of reduced TGFβ signaling (Fig. 2g and Supplementary Table 2)^28^. In contrast to the *daf-3(e1376)* null mutation, which abolished *daf-1*-dependent survivorship and is dauer formation defective (Daf-d), the *daf-3(syb2718)* allele obtained from our genetic screen behaved oppositely; it restored *hlh-30* survivorship and reproductive competence (Fig. 2h and Extended Data Fig. 2g), and on its own caused a dauer constitutive (Daf-c) phenotype, acting as a semi-dominant gain-of-function mutation (Extended Data Fig. 2h). Interestingly, the changed amino acid (p.Glu853Lys), falls within the MH2 domain (Extended Data Fig. 2f), and is predicted to disrupt inhibitory interactions with r-SMADs, uncoupling DAF-3/SMAD4 activity from upstream inputs^29^. Last, we tested *daf-12/VDR*, a major downstream transcriptional mediator of bile-acid-like steroid signaling essential for dauer formation^30^, but found that it was not required, consistent with both common and distinct regulators of resilience during larval dauer and ARD (Extended Data Fig. 2i).

### HLH-30 regulates TGFβ signaling in response to nutrient cues

**Fig. 3:**
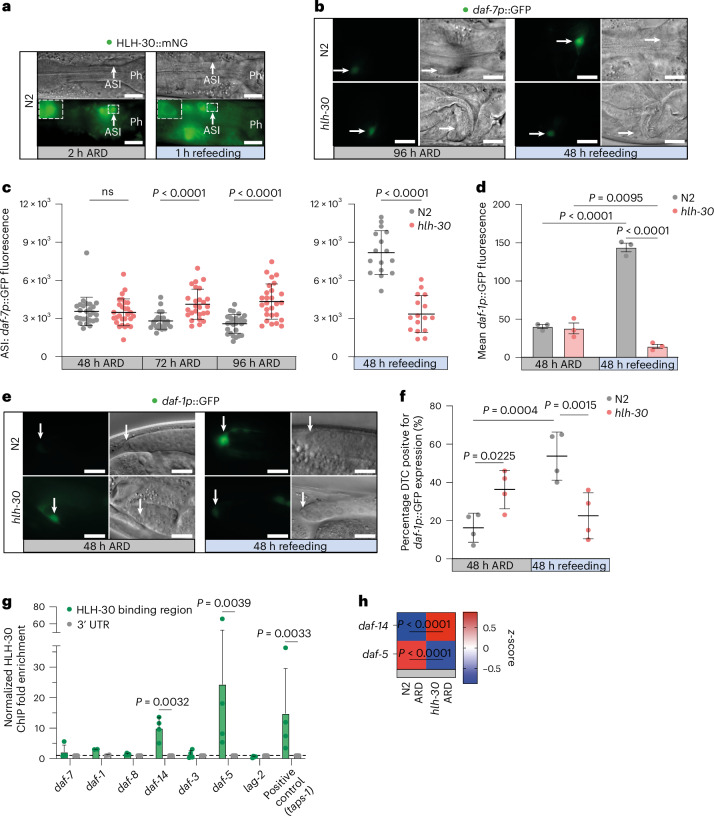
HLH-30 regulates TGFβ signaling in response to nutrient cues. **a**, *hlh-30::mNeonGreen* (*hlh-30::mNG*) expression pattern in ASI sensory neurons at 2 h of ARD and upon 1 h of refeeding 48-h ARD worms. Differential interference contrast (DIC) and fluorescence images of the head region. The arrow indicates an ASI neuron. Ph, terminal pharyngeal bulb. Scale bars, 10 μm. **b**, Representative images of *daf-7p*::GFP expression in the ASI neuron at 96 h of ARD and 48 h of refeeding after 96 h ARD. Fluorescence and DIC images of head regions. The arrow indicates an ASI neuron. Scale bars, 10 μm. **c**, Quantification of *daf-7p*::GFP expression in ASI neurons at 48, 72 and 96 h of ARD (80-ms exposure time) and after 48 h of refeeding (40 ms exposure time). Each dot represents *daf-7p*::GFP expression of one ASI neuron. BR = 3, one representative experiment shown. **d**, Quantification of whole-body *daf-1p::*GFP at 48 h of ARD and 48 h of refeeding measured with the COPAS Biosorter. Mean ± s.e.m., each dot represents one experiment. Two-way ANOVA followed by Fisher’s least significant difference post hoc test. **e**, Representative images of *daf-1p*::GFP expression in the DTC at 48 h of ARD and 48 h of refeeding. Fluorescence and DIC images of the distal gonad. The arrow indicates the DTC nucleus. Scale bars, 10 μm. **f**, Percentage of DTC positive for *daf-1p*::GFP expression of N2 and *hlh-30(tm1978)* at 48 h of ARD and after 48 h refeeding. BR = 4. One-tailed Mann–Whitney to compare the percentage of *daf-1*::GFP-positive DTC within ARD or refeeding and one-way ANOVA followed by Tukey’s post hoc test to compare genotypes across ARD and refeeding conditions. **g**, ChIP enrichment of HLH-30 binding to promotor regions of canonical TGFβ pathway genes. For each gene, one HLH-30 binding site (green) and the corresponding 3′ untranslated region (UTR) control (gray) was tested in this analysis. Data were normalized to 3′ UTR binding (dashed line)^66^. Mann–Whitney statistics (two sided) were performed on the non-normalized ∆∆c_t_ values. One dot represents one biological repeat. BR = 4. **h**, Normalized mRNA expression from RNA-seq data of *daf-14* and *daf-5* in 48-h ARD comparing WT and *hlh-30(tm1978)* mutants. Data and statistics are presented in Supplementary Table 2. If not stated otherwise, data are the mean ± s.d. Two-tailed Mann–Whitney test. Source data

**Extended Data Fig. 3:**
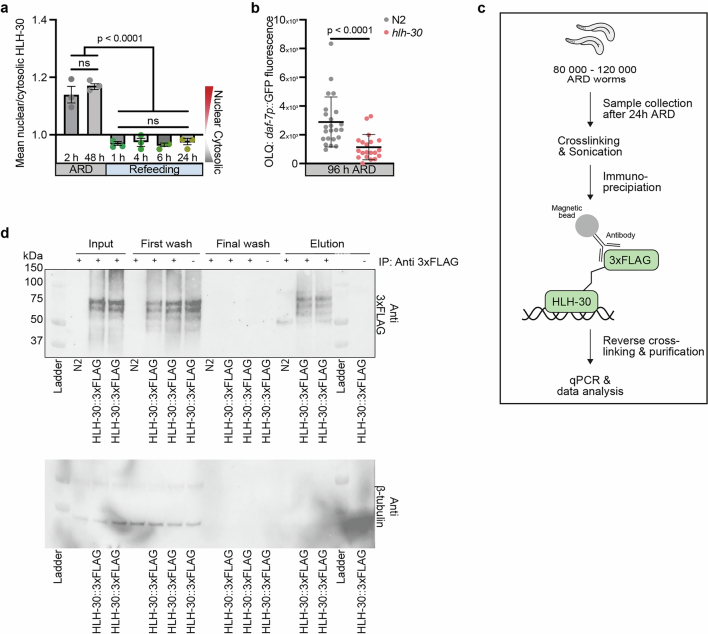
HLH-30 regulates TGFβ signaling in response to nutrient cues. **(a)** Nuclear/cytosolic *hlh-30::*mNeonGreen expression in ASI neurons under ARD (2 and 48 hours) and recovery from 48 h ARD (1, 4, 6, and 24 hours of refeeding). Each dot represents one experiment. BR = 3. Mean ± SEM **(b)** Quantification of *daf-7p*::GFP fluorescence in OLQ neurons of N2 and *hlh-30(tm1078)* at 96 hours of ARD. Exposure time 400 ms. Expression in OLQ is 5–10-fold lower than in ASI. Each dot represents the *daf-7p::*GFP expression in one OLQ neuron. BR = 3, one representative experiment. Mann-Whitney test (two-sided). **(c)** Schematic of ChIP-qPCR experimental design. **(d)** Control western blot ChIP-qPCR experiment showing specific elution of HLH-30::FLAG (BR = 2). ‘+’ indicates the use of an Anti-FLAG antibody during IP, ‘-‘ indicates no use of Anti-FLAG antibody (bait). Tubulin control staining was performed on the same gel after washing off the Anti-FLAG antibody. If not stated otherwise: Mean & s.d., One-way ANOVA followed by Turkey’s post-hoc test. Source data

The genetic epistasis experiments described above suggest that HLH-30(+) could act to downregulate TGFβ signaling under ARD. To directly address this idea, we measured HLH-30 nuclear localization and expression of *daf-7/*TGFβ ligand in the ASI neurons during ARD and refeeding. These neurons secrete TGFβ, serving as an endocrine source to regulate TGFβ receptor signaling in target tissues throughout the body^27,31^. We observed that HLH-30::mNeonGreen rapidly entered the nucleus of ASI neurons within 2 h of ARD induction, persisted during early ARD and promptly exited upon refeeding (Fig. 3a and Extended Data Fig. 3a). In line with nutrient-dependent regulation, *daf-7p::*GFP was down during WT ARD, but up upon refeeding (Fig. 3b,c). In *hlh-30* mutants, *daf-7p::*GFP dynamic regulation was the opposite: up during ARD and no response to refeeding (Fig. 3b,c). Interestingly, we also observed a low-level *daf-7p::*GFP expression in outer labial OLQ neurons under ARD, not seen under ad libitum conditions, and whose regulation was also perturbed by *hlh-30* (Extended Data Fig. 3b), suggesting ARD-specific regulation within these neurons. Together, our results indicate that HLH-30(+) partially downregulates *daf-7* expression in a cell-autonomous manner in the ASI neurons in response to nutrient cues.

We observed a similar regulatory pattern in WT whole-body *daf-1p*::GFP expression (Fig. 3d). In WT, global *daf-1p*::GFP was expressed at low levels during ARD, and became elevated upon refeeding. In *hlh-30* mutants, *daf-1p*::GFP was expressed at low levels similar to WT under ARD, but failed to upregulate upon refeeding, suggesting an inability to adapt to food cues (Fig. 3d). To examine tissue-specific regulation, we then monitored *daf-1p*::GFP within the DTC^32^. In WT, *daf-1p::*GFP expression in the DTC decreased during ARD and increased during refeeding (Fig. 3e,f). In *hlh-30* mutants, by contrast, mean *daf-1p*::GFP expression was elevated by 27% compared to WT during ARD, and did not respond to refeeding, showing a misregulation of *daf-1* receptor expression within the stem cell niche (Fig. 3e,f).

To test whether HLH-30 transcriptionally regulates TGFβ signaling components directly, we performed chromatin immunoprecipitation coupled with quantitative PCR (ChIP–qPCR) during ARD, pulling down HLH-30::FLAG and testing for candidate promoter enrichment on predicted HLH-30 binding sites by qPCR (Extended Data Fig. 3c,d). We found significantly enriched HLH-30 occupancy at *daf-14* and *daf-5* promoters (Fig. 3g). Accordingly, we saw that the *hlh-30* mutation caused significant upregulation of *daf-14* and downregulation of *daf-5* mRNAs under ARD (Fig. 3h), confirming that HLH-30 directly inhibits TGFβ signaling under ARD by both repressing the positive arm and promoting the negative arm of the pathway. Our findings are also supported by existing yeast one-hybrid data^33^ and *hlh-30* ChIP–seq in the *daf-2(e1370)* background^34^. We did not observe enrichment at *daf-7* or *daf-1* promoters (Fig. 3g). This observation is presumably because regulation mainly takes place in single cells where occupancy may fall below the limit of detection, binding might occur outside the tested region or regulation is indirect. The prominent rescue of *hlh-30* ARD lifespan collapse and other features by *daf-7/TGFβ* or *daf-1/TGFβRI* mutation, together with the deregulated transgene expression, suggest an integral role for HLH-30 regulating the TGFβ pathway at multiple points, starting from the ASI neuron and signaling throughout the entire body via a systemic mechanism.

### Regulation of growth signaling protects against a senescence-like state

**Fig. 4:**
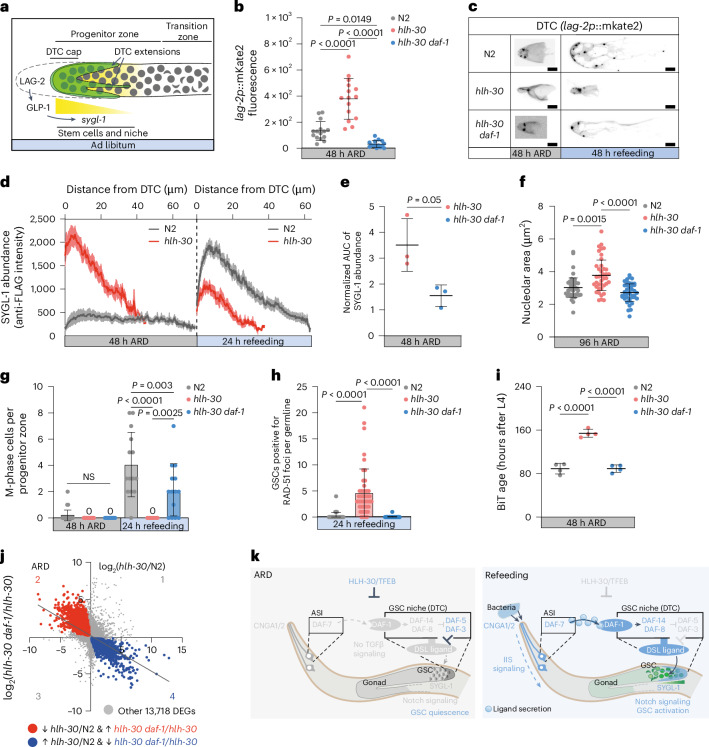
Regulation of growth signaling protects against a senescence-like state. **a**, Hermaphrodite distal gonad schematic. Germ cells in gray. The progenitor zone contains a distal pool of stem cells (area of *sygl-1* expression, yellow) and a proximal pool of differentiating cells. Forward growth signaling induces LAG-2 secretion from the DTC to GSCs, initiating mitotic cell division and *sygl-1* expression via GLP-1. SYGL-1 abundance declines with increasing distance to the DTC. **b**, Quantification of *lag-2p::mKate2::PH* expression intensity in the DTC cap at 48 h of ARD. Each dot represents *lag-2p::mKate2::PH* expression in one DTC per worm. **c**, *lag-2p::mKate2::PH* expression in DTC at 48 h of ARD and after 48 h refeeding. Fluorescence images. Contrast and intensity were adjusted to visualize DTC processes. Scale bars, 10 μm. **d**, Quantification of SYGL-1 abundance in the distal gonad of 48-h-ARD worms and of 24-h refed worms, based on intensity of α-FLAG staining. Average intensity values (*y* axis) were plotted against distance (μm) from the DTC (*x* axis). Lines indicate mean intensity; shaded areas are the s.e.m.; BR = 3, one representative experiment. **e**, Quantification of the area under the curve (AUC) of SYGL-1 expression. AUCs were normalized to the N2 AUC for each biological replicate. One-tailed Mann–Whitney test. BR = 3. **f**, Quantification of germ cell nucleolar area at 96 h of ARD. Each dot represents the nucleolar area in μm^2^ of one germ cell per worm. BR = 3, one representative experiment. **g**, Number of M-phase cells in the distal gonad arms (progenitor zone) at 48 h of ARD and 24 h of refeeding. BR = 3, one representative experiment. One dot indicates the number of M-phase-positive cells per gonad arm. **h**, Quantification of GSCs positive for RAD-51 foci in the *sygl-1*-positve area per gonad. One dot represents the number of positive GSCs per gonad arm per worm. Pooled from three BRs. **i**, Biological age prediction (BiT) from transcriptomes at 48 h of ARD. Each point represents one replicate. **j**, Correlation plot of *hlh-30(tm1978)*/N2 and *hlh-30 daf-1(m40)*/*hlh-30* DEGs at 48 h of ARD. Significantly regulated genes (adjusted *P* < 0.05) highlighted in red (genes down in *hlh-30* and reversed by *daf-1*, 2,347 DEGs, quadrant 2) or blue (genes up in *hlh-30* and reversed by *daf-1*, 2,083 DEGs, quadrant 4). Simple linear regression line in gray. Equation *Y* = −0.48*X* + 0.13; *R*^2^ = 0.27. **k**, Working model. In WT ARD HLH-30/TFEB is active and downregulates TGFβ signaling at multiple levels. Consequently, Notch signaling is inhibited via LAG-2/DSL downregulation in the DTCs, resulting in GSC quiescence. Upon refeeding, HLH-30 becomes inactive, resulting in reactivation of TGFβ and downstream signaling. Subsequently, stem cell niche remodeling (DTC outgrowth) is initiated, GSCs proliferate and worms reproduce. cGMP and insulin–IGF signaling (IIS) work upstream or in parallel to TGFβ signaling. Data and statistics are presented in Supplementary Table 2. If not stated otherwise, data are the mean ± s.d. One-way ANOVA followed by Tukey’s post hoc test. Genotypes N2, *hlh-30(tm1978)*, *hlh-30 daf-1(m40)*. Source data

*C. elegans* GSCs are regulated primarily by Notch signaling, which signals from the DTC niche to the stem cell pool. The DTC itself forms a cap-like structure around the GSCs and extends long processes to expand the zone of activation^35,36^ (Fig. 4a). Under ad libitum conditions, the Delta/Serate/LAG-2 (DSL) ligand expressed in the DTC niche binds to the GLP-1/Notch receptor in adjacent GSCs, to promote expression of target genes including *sygl-1*, resulting in stem cell activation and mitosis, and preventing premature meiosis^37–39^.

**Extended Data Fig. 4:**
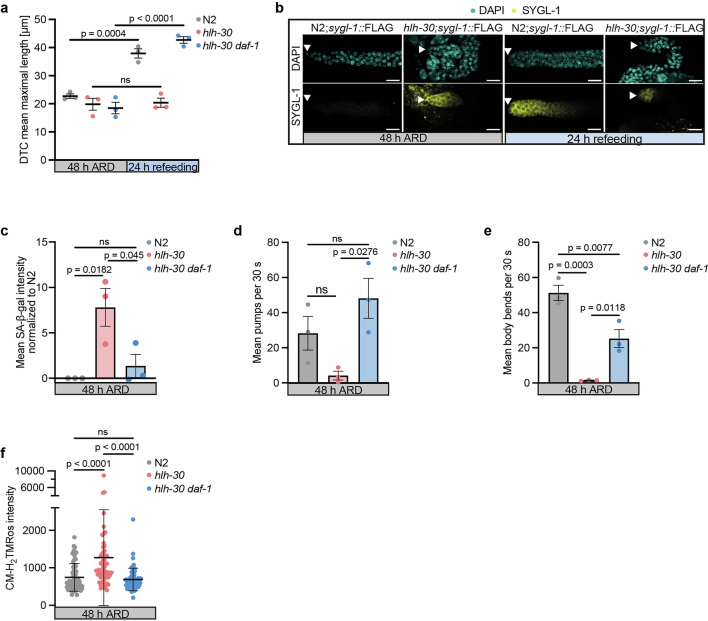
Regulation of growth signaling protects against senescence. **(a)** Distal tip cell (DTC) extension measured from the cap to the end of the most distal process containing *lag-2p*::mKate2::PH. Each dot represents the mean DTC length of one BR. **(b)** Representative images of SYGL-1 stained and DAIP labeled germline stem cells **(**GSCs) at 48 hours of ARD and 24 hours of refeeding. Images show a single z-layer of the germline. Genotypes N2 and *hlh-30(tm1978)*. Arrowhead show the distal end of the gonad. Scale bar 10 μm. **(c)** Mean SA-β-gal RGB color intensity in the head region of 48 h ARD worms normalized to N2. Genotypes N2, *hlh-30(tm1978)*, *hlh-30 daf-1(m40)*. Each dot represents the SA-β-gal mean intensity of one BR. **(d)** Mean pharyngeal pumping and **(e)** Mean body bending rates upon 48 hours of ARD. Genotypes: N2, *hlh-30(tm1978)*, *hlh-30 daf-1(m40)*. 30 second intervals. Each dot represents the mean pumping rate or mean body bends of one BR. **(f)** Quantification of mitochondrial ROS in N2, *hlh-30(tm1078), hlh-30 daf-1(m40)* at 48 hours of ARD. Pooled data from three BRs. Kruskal-Wallis followed by Dunn’s post-hoc test. If not stated otherwise: Mean & s.d., One-way ANOVA followed by Turkey’s post-hoc test. Source data

Given that TFEB–TGFβ impacts progeny production, we wondered whether it correspondingly affects Notch signaling and gonadal architecture and, thereby, GSC function during ARD and recovery. In particular, we hypothesized that in *hlh-30* mutants, increased TGFβ signaling from neurons to the DTC might result in derepression of Notch signaling during ARD and drive GSCs into a senescence-like state. If true, then *daf-1/TGFβ* mutation should result in a prominent rescue. We measured three parameters: *lag-2p::mKate2::PH* expression level in the DTC (niche), DTC long process extension (niche matrix remodeling) and *sygl-1::3xFlag* abundance (GSCs) as proxy measures of Notch activity. In WT, we observed that all three parameters were reduced under ARD (Fig. 4b–d), while DTC extension and *sygl-1::3xFlag* expression increased upon recovery, consistent with downregulation of Notch signaling in quiescence and upregulation during activation, respectively (Fig. 4c,d and Extended Data Fig. 4a,b). By contrast, *hlh-30* mutants exhibited elevated *lag-2p::mKate2::PH* and *sygl-1::3xFlag* expression under ARD compared to WT, yet during recovery, failed to extend DTC processes, and limited *sygl-1::3xFlag* expression, suggesting *hlh-30* loss dysregulates Notch signaling during both quiescence and activation in niche and stem cells (Fig. 4b–d and Extended Data Fig. 4a,b). Remarkably, *daf-1* mutation reversed features of *hlh-30*-induced Notch dysregulation (Fig. 4b,c,e and Extended Data Fig. 4a), implying that HLH-30 regulates Notch signaling via TGFβ to facilitate GSC resilience.

Of note, elevated Notch and TGFβ signaling are both considered hallmarks of senescent cells^40,41^, suggesting that their dysregulation in *hlh-30* mutants could contribute to a senescence-like state. In particular, we wondered if the *daf-1* mutation could protect against other *hlh-30*-induced senescence-like phenotypes, as described in Fig. 1. Indeed, *daf-1* loss was sufficient to restore GSC nucleolar area (Fig. 4f), mitosis (Fig. 4g), DNA damage status (Fig. 4h), transcriptomic age (Fig. 4i), SA-β-gal expression (Extended Data Fig. 4c), pumping activity, muscle health (Extended Data Fig. 4d,e) and mitochondrial ROS (Extended Data Fig. 4f) of *hlh-30* mutants.

**Extended Data Fig. 5:**
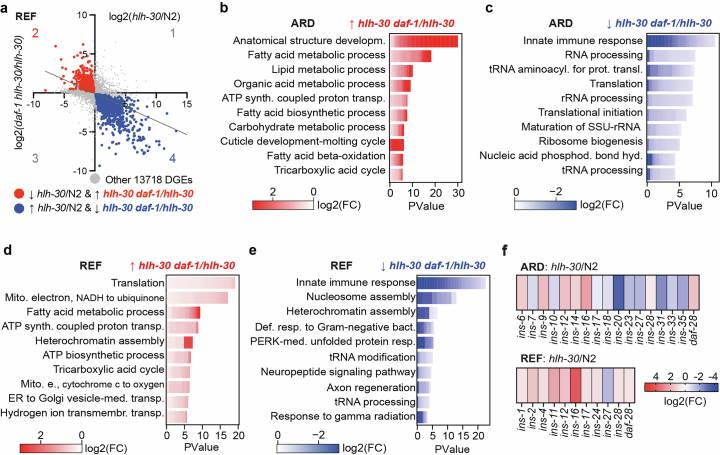
Downregulation of growth signaling partially reverses senescence transcriptome. **(a)** Correlation plot of differentially expressed genes (DEGs) of *hlh-30*/N2 and *hlh-30 daf-1*/*hlh-30* at 12 hours of refeeding (REF). DEGs (padj < 0.05) highlighted in red (genes down in *hlh-30* and reversed by *daf-1*, quadrant 2) or blue (genes up in *hlh-30* and reversed by *daf-1*, quadrant 4). Other DEGs, gray. Simple linear regression line in gray. Equation Y = −0.35X - 0.06; R^2^ = 0.27. **(b)** Gene ontology enrichment analysis of 2347 DEGs down in *hlh-30(tm1978)/*N2 ARD and up in *hlh-30 daf-1(m40)/hlh-30* ARD (padj < 0.05, quadrant 2). Top 10 biological processes of the DAVID GO BP DIRECT database. Complete GO term list is shown in Supplementary Table 3. Modified Fisher Exact p value, EASE Score. **(c)** Gene ontology enrichment analysis of 2083 DEGs up in *hlh-30/*N2 ARD and down in *hlh-30 daf-1/hlh-30* ARD (quadrant 4). Top 10 biological processes. Complete GO term list is shown in Supplementary Table 3. Modified Fisher Exact p value, EASE Score. **(d)** Gene ontology enrichment analysis of 1499 DEGs down in *hlh-30*/N2 and up in *hlh-30 daf-1*/*hlh-30* (padj<0.05, quadrant 2) at 12 hours of refeeding (REF). Top 10 biological processes. Modified Fisher Exact p value, EASE Score. **(e)** Gene ontology enrichment analysis of 2173 DEGs up in *hlh-30*/N2 and down in *hlh-30 daf-1/hlh-30* (padj<0.05, quadrant 4) at 12 hours of refeeding. Top 10 biological processes. Modified Fisher Exact p value, EASE Score. **(f)** Heat map of log2(FC) values of differentially expressed Insulin-like peptides in *hlh-30*/N2 at 48 hours of ARD and upon 12 hours of refeeding. padj< 0.05. Source data

Aside from physiological changes, we observed vast transcriptomic changes in *hlh-30* mutants compared to WT or *hlh-30 daf-1* during ARD and refeeding. Reasoning that genes downregulated in *hlh-30/*WT might be upregulated in *hlh-30 daf-1*/*hlh-30* and vice versa, we found a large fraction of such differentially expressed genes (DEGs) negatively correlated in ARD and refeeding (REF) (Fig. 4j and Extended Data Fig. 5a). We found mainly metabolic and growth processes misregulated in *hlh-30* and reversed in *hlh-30 daf-1* mutants (Extended Data Fig. 5b–e), highlighting the importance of the TFEB*–*TGFβ axis in metabolic remodeling during ARD. Notably, ARD DEGs strongly upregulated in *hlh-30/*WT and rescued by *daf-1* included the innate immune response, a feature similar to increased inflammatory signaling in senescence (Extended Data Fig. 5c). In accordance with increased RAD-51 foci observed in GSCs from *hlh-30* mutants, we could detect a signature of DNA damage response in our transcriptomic dataset (Supplementary Table 3). Interestingly, we found that *hlh-30* upregulated refeeding DEGs overlapped significantly with the SenMayo gene set for senescent cells (16/32 orthologs, *P* = 0.00068 by hypergeometric test; Supplementary Table 4), which are core genes associated with senescence^42^. Altogether, these findings suggest that the *hlh-30* mutation could trigger genomic instability, which, together with dysregulated metabolism, growth signaling and immune response, leads to a senescence-like state. Remarkably, this state can be prevented by downregulation of TGFβ signaling.

In summary, our results are consistent with the idea that ARD induces an aberrant cellular and organismal quiescence in *hlh-30* mutants that resemble cellular senescence, dependent on TGFβ signaling. Building on earlier work^27,32,39^, we incorporate HLH-30 and growth regulatory pathways into a working model: In response to ARD entry, HLH-30(+) normally downregulates TGFβ within ASI sensory neurons and TGFβ receptor in the DTC niche, resulting in lower TGFβ signaling and activation of the DAF-5–DAF-3 repressor complex (Fig. 4k). HLH-30 also directly downregulates *daf-14*/SMAD3 and upregulates *daf-5*/Sno-Ski, reinforcing the inhibition of the pathway. Consequently, downregulated *lag-2*/DSL in the niche reduces Notch signaling, resulting in reproductive quiescence. The proper regulation of this signaling cascade ensures stem cell resilience during dormancy and restoration of the stem cell pool upon refeeding. In *hlh-30* mutants, there is a misalignment between nutrient cues, and growth signaling regulation, TGFβ and Notch are improperly elevated during diapause, triggering a state reminiscent of mammalian senescence.

### TFEB controls resilience in mammalian diapause models

We next wondered whether the role of TFEB was conserved in mammalian diapause models, including mouse embryonic diapause and human cancer dormancy. These diapause states can be mimicked in vitro using INK128 a dual inhibitor of mTOR–PI3K^11,43^.

**Fig. 5:**
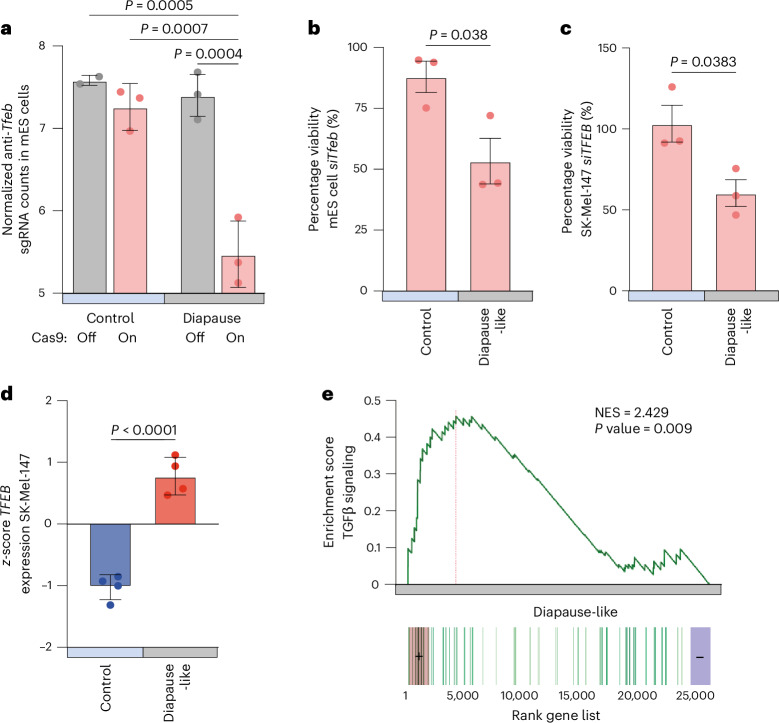
TFEB controls resilience in mammalian diapause models. **a**, sgRNAs counts for mammalian *TFEB* in the four analyzed conditions: control proliferating mES cells, control proliferating mES cells with doxycycline to induce the Cas9 activity, mES cells in diapause (by treatment with INK128) and mES cells in diapause treated with doxycycline. BR = 3. Two-way ANOVA followed by Tukey’s post hoc test. **b**, Effect of *TFEB* silencing on the viability of mES cells, when proliferating and in the diapause-like state. The diapause-like state was induced by treatment with INK128. Each column is normalized on its respective siRNA scrambled. BR = 3. Two-tailed unpaired *t*-test. **c**, Effect of *TFEB* silencing on the viability of SK-Mel-147 cells, when proliferating and in the diapause-like state. The diapause-like state was induced by treatment with INK128. Each column is normalized on its respective siRNA scrambled. BR = 3. Two-tailed unpaired *t*-test. **d**, *TFEB* expression levels from RNA-seq datasets of proliferating compared to diapause-like SK-Mel-147 cells. BR = 4. Two-tailed unpaired *t*-test. **e**, Gene-set enrichment analysis enrichment plot of the TGFβ signaling pathway genes in diapause-like SK-Mel-147 compared to proliferating SK-Mel-147. Statistical testing was performed using Kolmogorov–Smirnov-like statistics and adjusted for multiple testing using the Benjamini–Hochberg false discovery rate method. Data and statistics are presented in Supplementary Table 2. NES, normalized expression score. Source data

From a genome-wide CRISPR–Cas9 screen performed in mouse embryonic stem (mES) cells, we identified TFEB as a major determinant of diapause survival^44^. In particular, we used a doxycycline-inducible Cas9 system, which was activated in either proliferating control mES cells or in INK128-diapause mES cells, both carrying a genome-wide library of short-guide RNAs (sgRNAs)^45^. The relative abundance of sgRNAs was calculated as the ratio of before and after Cas9 induction and was analyzed for both diapause mES cells and proliferating mES cells 3 days after sgRNA editing. *TFEB* emerged as a gene depleted explicitly in the diapause population after Cas9 activation, suggesting a possible role in diapause survival and resilience (Fig. 5a)^44^. To confirm these findings under non-screening conditions, we targeted *TFEB* by short interfering RNA (siRNA)-mediated knockdown. Consistently, *TFEB* siRNA significantly reduced the survival of mES cells by 39.4% (Fig. 5b). These data indicate that TFEB is essential for the survival of mES cells in diapause but not for proliferating mES cells. Similarly, *hlh-30* mutation in *C. elegans* shows little effect on WT survival under ad libitum conditions but is essential for ARD^16,46^.

**Extended Data Fig. 6:**
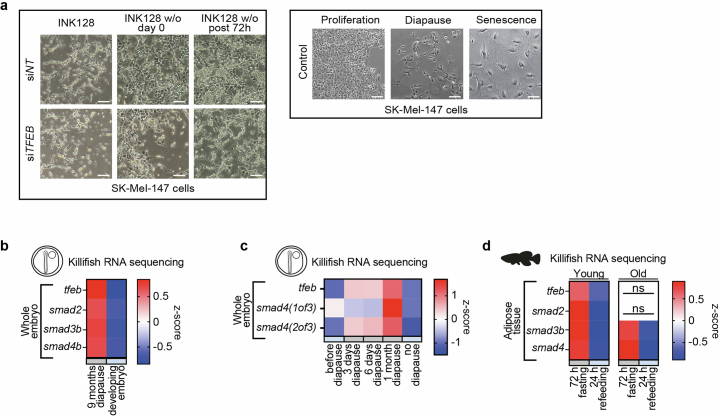
TFEB controls resilience in mammalian diapause models. **(a)** Representative images of SK-Mel-147 cells treated with si*TFEB* or si*NT* control in diapause, or 6 days post INK128 wash out (w/o) either at day 0 of siRNA treatment or 72 hours post siRNA transfection. Scale bar: 100 µm **(b)** Heat map of wild type killifish *tfeb, smad2, smad3b*, and *smad4b* expression from published RNA seq dataset at 9 months of diapause or developing embryos 6 days post fertilization^53^. z-score. BR = 4. **(c)** Heat map of wild type killifish *tfeb, smad4(1 and 2 of 3)* at 3 and 6 days and 1 months of diapause or developing embryos before and without diapause^54^. z-score. BR = 3. (**d)** Heat map of wild type killifish *tfeb, smad2, smad3b* and *smad4* expression from adipose tissues of young and old fasted and refed fish. RNA seq samples. z-score. BR = 4. ns = not significant. Source data

Having found evidence for a conserved role of *TFEB* in mammalian embryonic diapause, we asked if its function could be extended to the recently reported human cancer cell diapause-like state^11,12^. Cancer diapause is a resilience mechanism that enables tumor cells to evade chemotherapy and immune surveillance, only to emerge again and relapse. For these experiments, we used human melanoma SK-Mel-147 cells that undergo a robust and reversible diapause-like arrest in the presence of INK128 (ref. ^44^). Notably, siRNA-mediated downregulation of *TFEB* reduced survival of resistant cancer diapause-like cells by almost 50%, but did not affect survival of proliferating cancer cells (Fig. 5c). We wondered whether the remaining 50% of SK-Mel-147 cells that survived *TFEB* siRNA during the diapause state transitioned to senescence or continued to proliferate upon INK128 withdrawal. To test this, we investigated cellular morphology after 0 h and 72 h of drug removal after siRNA treatment. However, we did not detect senescence of SK-Mel-147 cells in the *siTFEB* or control cells; instead, the remaining viable cells proliferated slowly (Extended Data Fig. 6a). It is possible that the decision between senescence and cell death depends on cell type, media conditions or presence of niche cells. Interestingly, RNA sequencing (RNA-seq) revealed an upregulation in the levels of *TFEB* mRNA during diapause (Fig. 5d), and gene-set enrichment analysis revealed altered TGFβ signaling (Fig. 5e), further supporting the similarity of diapause-like states in human cancer cells and worms. It is important to note that elevated TGFβ signaling generally inhibits proliferation in mammalian cells^47^ but promotes proliferation in worms. Hence, in both systems, proliferation and growth are inhibited during diapause: in mammalian cells by activating TGFβ and in worms by inhibiting TGFβ.

## Discussion

In this work, we showcase ARD as a tractable model to illuminate the molecular architecture of stem cell longevity, regeneration, and senescence in vivo. Further, we develop a platform to decipher conserved resilience pathways governing diapause entry, maintenance and exit through rapid genetic screening.

We find that fasting-induced ARD entry downregulates growth signaling and fosters metabolic remodeling and reproductive quiescence, while refeeding during ARD exit reverses these processes, showing an adaptive response to nutrient cues that ensures stem cell and organismal resilience. This adaptive response is mediated by HLH-30/TFEB as its mutation causes a misalignment between nutrient cues and growth signaling, thereby inducing a senescence-like state that abrogates GSC and organismal longevity. In line with our results, *TFEB* overexpression in the mouse brain seems to mitigate senescence and memory deficits^48^. We speculate that mammalian senescence can also arise from a similar misalignment between nutrient supply, metabolism and growth signaling. Further study of *hlh-30*-induced dysfunction in non-proliferating cells, such as neurons, could provide a unique opportunity to define features of senescence in postmitotic cells.

Importantly, our studies establish a causal relationship between TFEB–TGFβ signaling axis regulation with respect to diapause and senescence. As part of this adaptive mechanism, we discovered that HLH-30 potently inhibits TGFβ signaling by acting at several points in the pathway. In the absence of HLH-30 during ARD, reinstated TGFβ regulation restores resilience during quiescence, protects against senescence and promotes rejuvenation during recovery, linking regulation and physiology in a causal chain. Interestingly, secreted mammalian TGFβ is often an integral component of the senescence-associated secretory phenotype ^49^. Future studies should illuminate the detailed cellular foci, regulatory circuity and time of action of HLH-30 function to convey cellular and organismal resilience. The fact that TGFβ receptor mutation alone leads to an ARD lifespan extension of WT (1.1–1.2-fold) could be because TGFβ growth signaling during ARD is downregulated but not off (Fig. 3). Additionally, *daf-1* loss might further attenuate ARD aging-induced cellular senescence phenotypes, thus extending worm life.

Notably, although HLH-30/TFEB is considered a master regulator of autophagy, surprisingly, we saw little specific impact of genetically disabling this process during ARD. Under prolonged fasting and hypometabolism, such as ARD, autophagy might be curtailed to prevent autolysis and ration molecular resources.

Besides TGFβ signaling, our screens also identified insulin–IGF signaling components and *tax-4*/CNGA1/CNGA2 as mediators of ARD survivorship and recovery. Both act within ciliated sensory neurons to regulate TGFβ^26^, and TAX-4 can additionally induce insulin-like peptide production^50^. In line with this, *hlh-30* loss causes dysregulation of numerous insulin-like peptides under ARD and refeeding (Extended Data Fig. 5f). Therefore, it seems likely that insulin–IGF signaling is also downregulated during ARD and upregulated during recovery, thus contributing to resilience and regeneration. Interestingly, reduced TGFβ, IIS and TAX-4 converge on DAF-16/FOXO to various extents, and HLH-30 and DAF-16 have overlapping target genes^34^. Further, DAF-16*/*FOXO is required for ARD survival^16^, and a critical downstream mediator of *daf-1 hlh-30* suppression (Fig. 2g), suggesting complementary roles of HLH-30*/*TFEB and DAF-16*/*FOXO in maintaining quiescence. Our study concurs with others linking TGFβ and insulin/IGF signaling to regulating Notch signaling within the GSC and niche^27,32,39^.

Extending our studies to the mammalian system, we find that *TFEB* knockdown alone was sufficient to drastically reduce the viability of dormant mES cells and resistant cancer cells in a diapause-like state by up to 50%. In both worm and mammalian cancer diapause models, TFEB and TGFβ components are highly regulated. In support of the role of TFEB in mammalian diapause states, recent work revealed that TFE3 (a TFEB family member) is recruited to dormancy loci during embryonic diapause^51^. Interestingly, studies in cell culture have revealed that TFEB regulates fat metabolism^52^ and preferentially binds FOXO1 (ref. ^34^), pointing toward a potential involvement of HLH-30/TFEB in lipid metabolism during diapause. Indeed, lipid metabolic pathways were prominently deregulated in our transcriptomics during *hlh-30* ARD (Extended Data Fig. 5b).

It is important to also point out that we observed clear differences between worms and the cancer diapause model. Whereas *hlh-30* mutants enter a senescence-like state upon refeeding, diapaused cells upon *TFEB* knockdown do not. Instead, they lose viability in diapause and have reduced regrowth after drug washout (Extended Data Fig. 6a). These differences in fate could reflect cell-type specificity or culture conditions because cancer diapause is induced with INK128 in nutrient-replete conditions, while worm diapause is induced under nutrient deprivation. Further, in the worm, stem or cancer cells are controlled by surrounding cells, a regulatory layer that is missing in vitro.

Turquoise killifish enter an obligate embryonic diapause as a resilience mechanism to outlast the dry season. Interestingly, *tfeb*, *smad4*, *smad2* and *smad3* are upregulated during killifish embryonic diapause based on existent transcriptome data (Extended Data Fig. 6b,c)^53,54^. We find that these same factors are also upregulated in the adipose tissue of fasted young adult fish and downregulated upon refeeding (Extended Data Fig. 6d). Some of these factors become misregulated in older fish, consistent with age-related perturbation of the fasting–feeding response^55^. It seems reasonable to speculate that loss of plasticity in older fish could reflect the accumulation of cells in a senescent state. Thus, rather than viewing ARD as a *C. elegans*-specific phenomenon, we argue that a core nutrient-sensing network is regulated and responsible for diapause and quiescent states across taxa, intimately linked to senescence.

The systemic regulation observed in *C. elegans* during ARD–refeeding and killifish fasting–refeeding paradigms may conceptually resemble heterochronic parabiosis experiments in mice, where circulating factors (for example, TGFβ relatives such as GDF15 and myostatin) modulate stem cell function across different physiological states^56,57^. While heterochronic parabiosis is often framed in terms of ‘youthful’ and ‘aged’ molecular environments, many of these systemic factors function through differential expression levels rather than an intrinsic aging signature. Similarly, plasma from exercised aged mice has been shown to transfer rejuvenating effects to sedentary aged mice, enhancing neurogenesis and cognitive function through liver-derived circulating factors such as Gpld1 (ref. ^58^). These findings suggest that systemic shifts in signaling—whether driven by age, exercise or metabolic states such as fasting–refeeding—can regulate resilience and regeneration across taxa.

In sum, our studies reveal the TFEB–TGFβ axis as a primordial circuit systemically regulating resilience, stem cell dynamics and senescence. Further study of ARD may provide unique molecular insights into the dynamics of senescence and its reversal.

## Methods

ARD induction was performed as previously described^16^. Briefly, worms were grown to the mid-L3 larval stage. Larvae were collected in M9 buffer and washed four times with M9 buffer. In total, 600 worms were pipetted onto 3-cm plates containing 4 ml nematode growth medium (NGM) with UltraPure agarose (Thermo Fisher Scientific) and 50 μg ml^−1^ ampicillin. Worms were maintained for their whole life on one plate at 20 °C, unless noted otherwise.

### Materials availability

Further information and requests for resources and reagents should be directed to and will be fulfilled by the corresponding author. This study did not generate unique reagents or new plasmids. Strains generated in this study are available upon request.

### Experimental model and subject details

Nematodes were cultured using standard techniques at 20 °C on NGM agar plates with the *Escherichia coli* strain OP50, unless otherwise noted. All strains used in this study are listed in the Table 1. Mutant strains were obtained from the Caenorhabditis Genetics Center (CGC) or the National BioResource Project and outcrossed to our N2 WT (*C. elegans* variant Bristol) as indicated in Table 1. CRISPR–Cas9 mutants were generated by SunyBiotech (https://www.sunybiotech.com/). ARD conditions are described below.

**Table 1:** Reagents

Reagent or resource	Source	Identifier
**Antibodies**		
Phospho-Histone H3 (Ser10) mouse monoclonal antibody	Cell Signaling	9706
Fluoromount-G	Invitrogen	00-4959-52
Goat serum	Cell Signaling	5425S
Anti-FLAG	Sigma-Aldrich	F3165
Alexa Fluor 488	Invitrogen	A-11008
Alexa Fluor	Invitrogen	594 A-11005
Anti-α-tubulin	Sigma-Aldrich	T9026
Anti-rabbit HRP	Invitrogen	G-21234
Anti-mouse HRP	Invitrogen	G-21040
**Bacterial strains**		
*E. coli*: strain OP50	CGC	WormBase: OP50
**Biological samples**		
SK-Mel-147	American Type Culture Collection	
KH2 ES cells	Oscar Fernandez-Capetillo’s laboratory	
**Chemicals, peptides and recombinant proteins**		
UltraPure agarose	Thermo Fisher Scientific	16500-500
MitoTracker Orange CM-H_2_TMROS	Thermo Fisher Scientific	M7511
C_1_-BODIPY 500/510 C_12_	Invitrogen	D3823
INK128 (sapanisertib)	SelleckChem	S2811
**Critical commercial assays**		
RNAeasy Mini Kit	QIAGEN	74104
NEBNext Poly(A) mRNA magnetics isolation module	New England Biolabs	E7490S
NEBNext Ultra Directional RNA Library Prep Kit for Illumina	New England	E7420S
Pierce 660-nm protein assay reagent	Thermo Fisher Scientific	1861426
Senescence β-galactosidase staining kit	Cell Signaling	9860
CellTiter-Glo luminescent cell viability assay	Promega	G7571
**Deposited data**		
RNA-seq data *C. elegans*	AA lab	NCBI GEO accession number: GSE291659
RNA-seq data killifish	AA lab	NCBI GEO accession number: GSE296348
**Experimental models: organisms–strains**		
*C. elegans*: strain N2 wild isolate.	CGC	WormBase: N2
*C. elegans*: *hlh-30(tm1978)* IV.	AA lab	Strain AA3658
*C. elegans*: *daf-1(m40)* IV.	CGC	Strain DR40
*C. elegans*: *daf-7(e1372)* III.	CGC	Strain CB1372
*C. elegans*: *pdk-1(sa680)* X.	CGC	Strain JT9609
*C. elegans*: *tax-4(p678)* III.	CGC	Strain PR678
*C. elegans*: *daf-2(e1368)* III.	AA lab	Strain AA4401
*C. elegans*: *daf-2(e1370)* III.	AA lab	Strain AA5311
*C. elegans*: *daf-3(syb2718)* X.	SunyBiotech	Strain PHX2718
*C. elegans*: *daf-3(syb2718)* X; *dpy-1(e1)* III.	AA lab	Strain AA5417
*C. elegans*: *daf-3(e1376)* X.	CGC	Strain CB1376
*C. elegans*: *daf-5(e1386)* II.	AA lab	Strain AA4397
*C. elegans*: *daf-16(mgDf50)* I.	AA lab	Strain AA60
*C. elegans*: *daf-1(m40) hlh-30(tm178)* IV.	AA lab	Strain AA5029
*C. elegans*: *daf-7(e1372)* III*; hlh-30(tm178)* IV.	AA lab	Strain AA5030
*C. elegans*: *pdk-1(sa680)* X; *hlh-30(tm1978)* IV.	AA lab	Strain AA5033
*C. elegans*: *tax-4(p678)* III; *hlh-30(tm1978)* IV.	AA lab	Strain AA5325
*C. elegans*: *daf-2(e1370)* III; *hlh-30(tm1978)* IV.	AA lab	Strain AA5450
*C. elegans*: *daf-3(e1376)* lll; *hlh-30(tm1978)* IV.	AA lab	Strain AA5339
*C. elegans*: *daf-3(syb2718)* X; *hlh-30(tm1978)* IV.	AA lab	Strain AA5252
*C. elegans*: *daf-12(rh61rh411)*X; *hlh-30(tm1978)* IV.	AA lab	Strain AA5289
*C. elegans*: *daf-1(m40) hlh-30(tm178)* IV*; daf-3(e1376)* X.	AA lab	Strain AA5034
*C. elegans*: *daf-1(m40) hlh-30(tm178)* IV*; daf-5(e1386)* II.	AA lab	Strain AA5036
*C. elegans*: *daf-1(m40) hlh-30(tm178)* IV*; daf-16(mgDf50)* I.	AA lab	Strain AA5035
*C. elegans*: *daf-1(m40) hlh-30(tm178)* IV*; daf-12(rh61rh411)*X.	AA lab	Strain AA5466
*C. elegans*: *daf-7(e1372)* III*; hlh-30(tm178)* IV*; daf-3(e1376)* X.	AA lab	Strain AA5037
*C. elegans*: *daf-7(e1372)* III*; hlh-30(tm178)* IV*; daf-5(e1386)* II.	AA lab	Strain AA5038
*C. elegans*: *mEx40[rol-6(su1006) daf-1p::GFP]*.	Riddle lab	Strain DR1767 *daf-1p::gfp*
*C. elegans*: *hlh-30(tm1978); mEx40[rol-6(su1006) daf-1p::GFP]*.	AA lab	Strain AA5553
*C. elegans*: *mIs7(daf-7p::GFP::daf-7 3«UTR*).	Riddle lab	Strain DR2021 *daf-7p::gfp*
*C. elegans*: *hlh-30(tm1978)* IV*; mIs7(daf-7p::GFP::daf-7 3«UTR)*.	AA lab	Strain AA4369
*C. elegans*: N2; *naIs37[pGC457 Plag-2(3kb)::mCherry-PH::let-858(3’); unc-119(+)]*.	Hubbard lab	Strain GC1038 *lag-2p::mCherry-PH*
*C. elegans*: *hlh-30(tm1978)* IV*; naIs37[pGC457 Plag-2(3kb)::mCherry-PH::let-858(3’); unc-119(+)]*.	AA lab	Strain AA5336
*C. elegans*: *daf-1(m40) hlh-30(tm1978)* IV*; naIs37[pGC457 Plag-2(3kb)::mCherry-PH::let-858(3’); unc-119(+)]*.	AA lab	Strain AA5338
*C. elegans*:*hlh-30(syb809[hlh-30::mNeonGreen]) IV*.	SunyBiotech	Strain PHX809 *hlh-30:: mNeonGreen*
*C. elegans*: CB1386.	CGC	Strain CB4856 Hawaiian strain
*C. elegans*: *hlh-30(tm1978)* in CB1386. 8 back crosses.	AA lab	Strain AA4398
*C. elegans*: *sygl-1(am307[3xFLAG::sygl-1]) I*.	CGC	Strain WU1770
*C. elegans*: *sygl-1(am307[3xFLAG::sygl-1]) I.; hlh-30(tm1978) IV.*	AA lab	Strain AA5415
*C. elegans*: *atg-7(bp411) IV.*	Melendez lab	Strain QU190
*C. elegans*: *lgg-2(tm5755) IV.*	AA lab	Strain AA4371
*C. elegans*: *foxSi27[pie-1p::tomm-20::mKate2::HA::tbb-2 3' UTR* *(oxti385)I].*	Zuryn lab	Strain SJZ106
*C. elegans*: *hlh-30/tm1978)IV;foxSi27[pie-1p::tomm-20::mKate2::HA::tbb-2 3' UTR* *(oxti385)I].*	AA lab	Strain AA5464
*C. elegans*: *him-5(e1490)* V.	CGC	Strain DR466
*C. elegans*: *unc-51(e369)* V.	AA lab	Strain AA3751
*C. elegans*: *epg-8(bp251) I;him-5(e1490)* V.	CGC	Strain HZ1691
*C. elegans: atg-13(bp414)* III.	CGC	Strain HZ1688
*C. elegans*: *epg-8(bp251) I;him-5(e1490)* V.	CGC	Strain HZ1691
*C. elegans*: *atg-9(bp564) him-5(e1490)* V.	CGC	Strain HZ1687
*C. elegans*: *him-5(e1490) V;atg-2(bp576)* X.	CGC	Strain HZ1683
*C. elegans*: *atg-7(bp411)* IV.	Lapierre lab	Strain QU190
*C. elegans*: *atg-5(bp546)* I.	Zhang lab	Strain HZ931
*C. elegans*: *K06A1.5(ok3224)* II.	CGC	Strain RB2372
*C. elegans*: *lgg-2(tm5755)* IV.	AA lab	Strain AA4371
*C. elegans*: *atg-3(bp412) IV;him-5(e1490)* V.	Zhang lab	Strain HZ903
*C. elegans*: *vps-18(syb773)* II.	SunyBiotech	Strain PHX773
*C. elegans*: *vha-2(ok619)* III.	CGC	Strain RB807
*C. elegans*: *vha-3(ok1501)* IV.	CGC	Strain VC1003
*C. elegans*: *vha-12(ok821)* X.	CGC	Strain RB938
Killifish (*Nothobranchius furzeri*)	AA lab	Strain GRZ-AD
**Software and algorithms**		
Flaski	Bioinformatics Core Facility of the Max Planck Institute for Biology of Ageing	NA
Galaxy platform	https://galaxyproject.org/	NA
MiModD	http://www.celegans.de/en/mimodd	
LAS X	Leica Microsystems	NA
Zen 2.3	Zeiss Microscopy	NA
Flow Pilot	Union Biometrica	NA
ImageJ	National Institutes of Health	NA
Imaris	Oxford instruments	NA
Photoshop and Illustrator	Adobe	NA
Excel	Microsoft	NA
Prism	GraphPad	NA
R (survival)	^81^	NA
Flexbar v2.5	^82^	NA
HISAT v0.1.6-beta	^83^	NA
StringTie v1.04	^84^	NA
Cufflinks v2.2.1	^85^	NA
**Other**		
SunyBiotech: CRISPR–Cas9 mutants	https://www.sunybiotech.com/	
**qPCR primer**		
*daf-7*, Fwd	CTATTAGGGGCCAATTCCGCA	qPCR
*daf-7*, Fwd	GAGGCGGGCTCTCATATCCA	qPCR
*daf-7 3*′ UTR, Fwd	GAGGCGGGCTCTCATATCCA	qPCR
*daf-7 3*′ UTR, Rev	TCGAAGGAGGTAGTGGAAGGA	qPCR
*daf-1*, Fwd	ACAATAAAAGAAAGTCACGTGGCAA	qPCR
*daf-1*, Fwd	ACAATAAAAGAAAGTCACGTGGCAA	qPCR
*daf-1* 3′ UTR, Fwd	TCGAAGGAGGTAGTGGAAGGA	qPCR
*daf-1* 3′ UTR, Rev	CTGAGGTACACGGTGAGGAG	qPCR
*daf-8*, Fwd	TCAGGTGAGCTACTCAAATGGG	qPCR
*daf-8*, Fwd	TCATACCAGTTTTCATTCCGAGTT	qPCR
*daf-8* 3′ UTR, Fwd	TGCGTGTGATCAATTTTGTTGATAG	qPCR
*daf-8* 3′ UTR, Rev	TCGAGCAGTTCTAAAGCCCTC	qPCR
*daf-14*, Fwd	TCGATCACGTGCTATCGTTCC	qPCR
*daf-14*, Fwd	ACGTGAGCTTCTGCTCCTAAC	qPCR
*daf-14* 3′ UTR, Fwd	CGAATGCGTACCCGTTTGAT	qPCR
*daf-14* 3′ UTR, Rev	AAGCATTCAAGAGACTGGGAA	qPCR
*daf-3* P1, Fwd	TGGAAAAGGGCGAGAGAGAG	qPCR
*daf-3* P1, Fwd	AGTCAAGATATGCGCAACGG	qPCR
*daf-3* P2, Fwd	ACCGAGGTCATGTGCACTCT	qPCR
*daf-3* P2, Fwd	ACCGAGGTCATGTGCACTCT	qPCR
*daf-3* 3′ UTR, Fwd	GTTCATTGTGAGCTTTGAGCTGT	qPCR
*daf-3* 3′ UTR, Rev	GGGCCACGGTGCAATATATGA	qPCR
*lag-2*, Fwd	GCGCAATCGAAACAACAGGT	qPCR
*lag-2*, Rev	CAAGACCCCGATGCGGTAAT	qPCR
*lag-2* 3′ UTR, Fwd	GTGTGCTTCTTCCACGAGTA	qPCR
*lag-2* 3′ UTR, Rev	GGGAGATGGGACGGAATGTA	qPCR
*taps-1*, Fwd	CACTTCTCATACACTCCGCCA	qPCR
*taps-1*, Rev	GCATGACCTGAGCACGAGA	qPCR
*taps-1* 3′ UTR, Fwd	CCGGGGTTTGAATGCGGAAAT	qPCR
*taps-1* 3′ UTR, Rev	CTTCAGCGGAGGATTGAGAGAG	qPCR

AA lab, Adam Antebi lab; Fwd, forward; GEO, Gene Expression Omnibus; NA, not applicable; Rev, reverse.

### ARD induction

ARD induction was performed as previously described^16^. Briefly, worms were grown to the mid-L3 larval stage. Larvae were collected in M9 buffer and washed four times with M9 buffer. In total, 600 worms were pipetted onto 3-cm plates containing 4 ml NGM with UltraPure agarose (Thermo Fisher Scientific) and 50 μg ml^−1^ ampicillin. Worms were maintained for their whole life on one plate at 20 °C, unless noted otherwise.

### Recovery from ARD

ARD worms were transferred to NGM plates seeded with *E. coli* OP50. Successful exit and recovery from ARD were determined by visual inspection (body size, motility) and the ability to produce progeny, indicating germline reconstitution. For total brood size measurement, individual ARD worms were picked to OP50-seeded 3-cm NGM plates, and the progeny number per worm was counted. While producing progeny, the worms were transferred regularly until reproduction had ceased.

### Lifespan experiments

ARD lifespans were determined by scoring a population of about 400–600 ARD worms every third day. In one experiment several plates per genotype were scored. Day 0 corresponds to the L3 stage^16^. Lifespan experiments under ad libitum conditions were performed as described^59^. Day 0 corresponds to the L4 stage.

### *hlh-30* suppressor screen

*hlh-30(tm1978)* L4 larvae were mutagenized with 0.5% EMS in M9 buffer according to standard protocols^60^. Each 10 mutagenized P0 adults were allowed to lay eggs overnight on a 10-cm plate. F1 adults were treated with hypochlorite to enrich eggs, and ARD was induced on mid-L3 worms of the F2 generation (see above). After 20 days in ARD, *hlh-30* suppressors were transferred to OP50-seeded plates and recovery was monitored (‘Recovery from ARD’). Approximately 24,000 genomes were screened. Reproductive worms were singled, resulting in 15 strains after retesting: EMS-1 and EMS-7 (test mutagenesis), A1, I4, 37, 39, 40, 43, 51, 67, 70, 71, 87, 104 and 109 (mutagenesis 2).

### Whole-genome sequencing and Galaxy MiModD analysis

For genomic sequencing, we prepared genomic DNA from strains listed above with QIAGEN Gentra PureGene tissue kit. Sequence libraries were created using the TruSeq DNA sample prep (Illumina). Libraries were sequenced on a HiSeq 2500 (Illumina) to generate 150-bp paired-end reads. Library preparation and sequencing were performed by the Max Planck Genome Center. Sequencing data were analyzed using Galaxy software. The WS220/ce10 *C. elegans* assembly was used as the reference genome for annotation.

Single nucleotide polymorphism-based mapping was performed on mutant strains A1, 39, 40, 43, 71 and 109 by crossing them to *hlh-30(tm1978)* in the Hawaiian strain CB4856 background. F1 generation was bleached and ARD was induced on the F2 generation. After 20 days, ARD worms were transferred to OP50-seeded plates, recovered worms were singled on 6-cm plates, and progeny were pooled for genomic DNA preparation. Pooled DNA was sequenced on an Illumina HiSeq platform (paired-end 150 nucleotide). Mutations were identified with MiModD software (https://celegans.biologie.uni-freiburg.de/?page_id=917/). WS220/ce10 *C. elegans* assembly was used as a reference genome for annotation. Causative mutations were confirmed by testing existing reference alleles or CRISPR–Cas9-designed base-pair change of identified genes (that is, *daf-3(syb2718)* in the *hlh-30* background on ARD recovery).

### Dauer assay

To determine dauer formation, synchronized worm populations were generated by allowing 20 worms to lay eggs for 4 h. Eggs were incubated at 25 °C for 48 h. Dauer characteristics such as a constricted pharynx, dark coloration and a long, thin body shape were used for identification. To study *daf-3(syb2718)* dominant or recessive dauer entry, we used a recessive *dpy-1(e1)* marker to score for heterozygosity. *daf-3(syb2718);dpy-1* and control worms were crossed with WT males. F1 cross progeny eggs were shifted to 25 °C for 48 h, and heterozygous worms (non-dumpy phenotype) were scored for dauer entry.

### Worm size measurements

Images of worms were taken with a Zeiss Axio Imager Z1 or a Leica M165 FC microscope. Body length was determined using ImageJ. At least 25 worms were analyzed per genotype.

### RNA-seq *C. elegans*

For RNA-seq, total RNA was prepared from at least 3,000 worms per genotype, using the RNeasy Mini Kit (QIAGEN). Four independent biological replicates were prepared. polyA^+^ mRNA was isolated using NEBNext Poly(A) mRNA Magnetics Isolation Module (New England Biolabs). RNA-seq libraries were prepared with the NEBNext Ultra Directional RNA Library Prep Kit for Illumina (New England Biolabs). Libraries were quantified by fluorometry, immobilized and processed onto a flow cell with a cBot (Illumina) followed by sequencing-by-synthesis with TruSeq v3 chemistry on a HiSeq 2500 at the Max Planck Genome Center. Reads were quality trimmed with Flexbar v.2.5, then mapped to the reference genome (WBcel235.80) using HISAT2 v.2.0.4. Respective assemblies were merged with cuffmerge v.2.2.1, and differential gene expression analysis was performed with Cuffquant v.2.2.1 and Cuffdiff v.2.2.1. Gene Ontology annotation and enrichment was performed using DAVID bioinformatics resource database analysis via the Flaski web app for data analysis and visualization^61^ developed by the Bioinformatics Core Facility of the Max Planck Institute for Biology of Ageing.

### RNA-seq killifish

All experiments were performed on adult (young 6–8 weeks, and old 18–20 weeks old) African turquoise killifish *Nothobranchius furzeri* laboratory strain GRZ-AD. Adult fish were single housed in 2.8 l tanks from the second week of life. Water parameters included a pH of 7–8, a kH of 3–5 and a temperature of 27.5 °C, 12 h of light and 12 h of darkness and fed with 10 mg of the dry pellet (BioMar INICIO Plus G) and Premium Artemia Coppens twice a day. The fish were either fasted for 72 h or fasted for the same amount of time and refed for 24 h and then euthanized. To reduce variability due to circadian rhythms, fish were euthanized all at once within 2 h in the early afternoon. Harvested tissues were snap frozen in liquid nitrogen and stored at −80 °C. RNA extraction of all samples was done at the same time. 1 μg of total RNA was used for library preparation. The sequencing was performed on the Illumina HiSeq 4000 sequencing system (∼50 million reads per sample) using a paired-end two × 100-nucleoide-long sequencing protocol. After removing rRNA and tRNAs, reads were pseudo-aligned to the reference genome (Nfu_20140520) using Kallisto (0.45.0). Pairwise differential gene expression was performed using DESeq2 (1.24.0). Animal experimentation was approved by ‘Landesamt für Natur, Umwelt und Verbraucherschutz Nordrhein-Westfalen’: 81-02.04.2019.A055.

### Biological age prediction of worms

Biological age prediction of worms was carried out using a transcriptome-based aging clock, BiT age^25^. Source code was downloaded from https://github.com/Meyer-DH/AgingClock/. Counts-per-million normalized RNA-seq reads for each genotype were used as input for BiT age analysis.

### SenMayo gene analysis in *C. elegans*

We transferred the 125 SenMayo genes^42^ to *C. elegans* orthologs using OrthoList 2 (ref. ^62^). If there were multiple orthologs, we chose the one with the most matches in homology databases. In total, we generated a list of 32 *C. elegans* genes (SenMayo *C. elegans* orthologs; Supplementary Table 4). We used FLASKI^61^ to perform hypergeometric testing (population size 20,000) comparing the SenMayo *C. elegans* gene set with gene sets up or down in *hlh-30*/N2 upon refeeding (REF).

### Imaging and image analysis

For live imaging, worms were anesthetized, if not stated differently, in 0.1% sodium azide. Image analysis was performed on two-dimensional (2D) or 3D images using Fiji software^63^. *hlh-30::mNeonGreen* nuclear localization in ASI neurons was determined at 2 h and 48 h of ARD, and after refeeding 48-h ARD worms for 1, 4, 6 or 24 h using a Zeiss Axio Imager Z1 microscope. Nuclear localization ratios were determined by measuring the fluorescence intensity in the nuclear and the cytosolic region of the ASI neurons. *daf-7p::gfp* expression was determined at 48, 72 and 96 h of ARD and 48 h of refeeding after a 48-h ARD period in one ASI neuron per worm using a Zeiss Axio Imager Z1 microscope. *daf-7p::gfp* expression in the OLQ was determined after 96 h of ARD. *daf-1p::gfp* expression in the DTC was determined at 48 h of ARD and 48 h of refeeding 48-h ARD worms using a Zeiss Axio Imager Z1 microscope. Whole-body *daf-1p::gfp* expression was monitored with the COPAS Biosorter (Union Biometrica, setting: green 450). *lag-2p::mCherry-PH* expression was determined in 48-h-old ARD worms. Worms were imaged with the Leica SP8 confocal microscope. *z*-stacks of whole DTCs (*z*-stack size, 0.3 µm) were captured. The sum projection of the DTC *z*-stacks was used to measure the total fluorescence intensity of the DTC cap structure. Maximal DTC length was determined by measuring the length of the longest DTC extension from distal to proximal using the *lag-2p::mCherry-PH* construct to color the DTC. GSC nucleolar area was determined from photos of the gonad taken with a Zeiss Axio Imager Z1 microscope (DIC contrast). Per genotype, 15 gonad arms from different animals were analyzed, scoring the area of each 3–5 nuclei located in the vicinity of the DTC. GSC mitochondria sphericity was assessed using *tomm-20::mKate2* expression under a germline-specific promotor (*pie-1*) in 48-h-old ARD worms and worms refed from a 48-h ARD period for 48 h. Worms were anesthetized in levamisole and imaged with the Leica SP8-X confocal microscope. *z*-stacks of whole gonad arms (*z*-stack size, 0.35 µm) were captured. Image analysis was performed in 3D using the mitochondria analyzer plugin in Fiji^64^ in the GSC (most distal 30 µm per gonad arm, thresholding method: mean). For different immunofluorescence analysis, germline dissections and staining were performed as described previously^65^. Briefly, ARD and refed ARD worms were anesthetized in 200 mM levamisole, dissected, fixed in 2% formaldehyde and post-fixed in 100% methanol at −20 °C for 10 min. Fixates were blocked in 30% Goat serum (Cell Signaling, 5425S) or 1% BSA and stained with different primary antibody (Phospho-Histone H3, 1:150 dilution, Cell Signaling, 9706S; anti-FLAG, 1:1,000 dilution, Sigma-Aldrich, F3165, anti-RAD-51, 1:1,000 dilution, a gift from the Smolikove lab) overnight. Secondary antibody staining (Alexa Fluor 488, A-11008, Alexa Fluor 594, A-11005, 1:400 dilution) was followed by fixate embedding in Fluoromount-G (Invitrogen, 00-4959-52) containing DAPI. *z*-stacks of whole gonad arms (*z*-stack size, 0.3 µm) were captured using a Leica SP8-X or a Leica SP8-DLS confocal microscope. Imaging analysis was performed on *z*-projections in Fiji^63^ and for the cell cycle analysis using the 3D image viewer of Imaris.

### SA-β-gal assay

Equal amounts of 48-h-old ARD worms were freeze-cracked and stained using a senescence β-galactosidase staining kit (Cell Signaling, 9860) according to the manufacturer’s instructions. Image analysis was performed in Fiji^63^, and color values were generated on RGB images.

### CM-H_2_TMROS staining

ARD plates contained 2 µM MitoTracker CM-H_2_TMRos (Thermo Fisher Scientific). After 48 h in ARD, worms were transferred to a plate without the dye for 1 h to remove traces of air-oxidized dye. Worms were anesthetized using levamisole and imaged using the Zeiss Axio Imager Z1 microscope. Image analysis was performed in Fijij. The whole body was analyzed for orange fluorescence, excluding the head and tail regions.

### ChIP–qPCR

ChIP–qPCR was performed after one day of ARD, as described previously^66^. Briefly, at mid-L3 stage, 80,000–120,000 worms were induced into ARD and cultured on 10-cm ARD plates. Two genotypes were included: HLH-30::3xFLAG and WT. After 24 h of ARD, worms were collected in M9 buffer, snap frozen in liquid nitrogen, and stored at −80 °C.

Once two independent biological repeats were collected, samples were thawed and incubated with 2% paraformaldehyde solution for 20 min at room temperature. A total of four biological replicates were used. Worms were then washed with HLB buffer (50 mM HEPES-KOH, 150 mM NaCl, 1 mM EDTA, 0.1% sodium deoxycholate, 0.1% SDS and cOmplete Protease Cocktail (Roche)) and sonicated with the Bioruptor Plus (Diagenode) (three times, five cycles with 30 s on and 30 s off at 4 °C, low power). The total extract was centrifuged (20,000*g*, 10 min, 4 °C), and the supernatant transferred to a new microcentrifuge tube. Protein concentration was measured using a Bradford assay (Pierce Coomassie Plus, Bradford-Assay).

The total protein load was used for HLH-30::3xFLAG immunoprecipitation. Three input samples were saved and flash frozen in liquid nitrogen for later analysis. Total extract was incubated with Dynabeads Protein G magnetic beads (Thermo Fisher Scientific) for 1 h at 4 °C to remove unspecific binding proteins. 1 µl of anti-FLAG antibody (Sigma-Aldrich) was incubated with the total extract overnight. One ‘bait’ immunoprecipitation without antibody was performed for each biological repeat. Next, 50 µl magnetic beads were added to the lysate–antibody mixture for 2 h at 4 °C. Bead conjugates were washed four times (2× WB1 (50 mM HEPES-KOH, 150 mM NaCl, 1 mM EDTA, 0.1% Triton X-100, 0.1% SDS, 1 mM PMSF), 1× WB2 (50 mM HEPES-KOH, 500 mM NaCl, 1 mM EDTA, 0.1%Triton X-100, 0.1% SDS, 1 mM PMSF), 1× WB3 (50 mM HEPES-KOH, 0.25 mM LiCl, 1 mM EDTA, 0.5% sodium deoxycholate, 0.5% NP-40)). Washing solutions were incubated for 5 min at 4 °C on a rotator shaker. Antibody conjugates were eluted from the magnetic beads by adding the elution buffer (50 mM Tris-HCL, 1% SDS, 10 mM EDTA) and incubation for 20 min at 65 °C.

For the following steps, one input sample per biological replicate was thawed and treated equally to immunoprecipitation samples. Samples were treated with 1 µl of 10 mg ml^−1^ RNase A (1 h, at 36 °C) and followed by 1 µl of 10 mg ml^−1^ Proteinase K treatment (2 h, at 55 °C). Reverse crosslinking was performed overnight at 65 °C. DNA was purified with phenol–chloroform–isoamylalcohol extraction. Total input DNA and immunoprecipitation DNA was loaded for real-time qPCR. Primer, DNA, and Power SYBR Green Master Mix were pipetted using the JANUS automated workstation (PerkinElmer). Four technical replicates were transferred to the 384-well plate per sample. Before the experiment, all primer sets were validated using standard dilution curves (primer efficiencies were between 90% and 110%). The comprehensive primer list can be found in Table 1. Real-time qPCR was performed using the ViiA 7 Real-Time PCR system machine (Applied Biosystems). ∆*c*_t_ and ∆∆*c*_t_ values were calculated as described previously^66^. ∆*c*_t_ = *c*_t_ (immunoprecipitation sample) − *c*_t_ (input sample); ∆∆*c*_t_ = ∆*c*_t_ (immunoprecipitation sample) − ∆*c*_t_ (control immunoprecipitation sample). The fold change was calculated using 2^−^^∆∆ct^.

### Western blotting

For western blotting, the following ChIP samples were used: input (worm lysis), first wash after immunoprecipitation, final wash and sample elution. Samples were directly added to 4× Laemmli buffer with 0.9% 2-mercaptoethanol and incubated for 5 min at 95 °C. Next, protein samples were loaded on a midi protein gel (Bio-Rad). The gel was blotted to a midi nitrocellulose membrane (Bio-Rad) using the Trans-Blot Turbo Transfer System (Bio-Rad). The membrane was blocked for 1 h in 5% skim milk and incubated with primary anti-FLAG antibody overnight at 4 °C. Next, the membrane was incubated with the secondary antibody for 2 h at room temperature and imaged using Western Lightning Plus Enhanced Chemiluminescence Substrate. The following antibodies were used: anti-FLAG, 1:1,000 dilution, anti-α-tubulin (Sigma, T9026; 1:10,000 dilution) Sigma-Aldrich, F3165, anti-rabbit horseradish peroxidase (HRP; Thermo Fisher, G-21234; 1:5,000 dilution), anti-rabbit HRP (Thermo Fisher, G-21234; 1:5,000 dilution) and anti-mouse HRP (Thermo Fisher, G-21040; 1:5,000 dilution).

### Cell culture procedure

ES cells were grown on gelatin 1% with ES medium (EM): Dulbecco’s modified Eagle’s medium (DMEM, high glucose, Life Technologies) supplemented with 15% fetal bovine serum (FBS, Gibco), penicillin–streptomycin (100 U ml^−1^), LIF (1,000 U ml^−1^), 0.1 mM non-essential amino acids, 1% glutamax and 55 mM β-mercaptoethanol. Diapause was induced by 200 nM INK128 (S2811, SelleckChem). Human melanoma SK-Mel-147 cells were grown in DMEM (high glucose, Life Technologies) supplemented with 10% fetal bovine serum (FBS, Gibco) and penicillin–streptomycin (100 U ml^−1^). The diapause-like state was induced by 100 nM INK128.

### Genome-wide sgRNA screening

To perform the genome-wide screen, we used a CRISPR–Cas9 system previously described^45^. ES cells were generated from mice ubiquitously expressing Cas9 under control of the endogenous *Cola1a* locus and a tetracycline responsive operator transgene; reverse tetracycline-controlled transactivator synthesis is under the control of the endogenous *ROSA26* locus. ES cells from these mice were infected with a lentiviral library encoding sgRNAs targeting 19,150 mouse genes, with approximately five independent sgRNAs per gene. Addition of doxycycline to the ES cells causes inducible expression of the Cas9 enzyme, which induces editing of the sgRNA targets. Diapause was induced in ES cells through the mTOR–PI3K inhibitor INK128 at 200 nM. After 5 days, half of the proliferating cells and half of the diapaused cells were sequenced to know the initial diversity of the sgRNA library. Doxycycline was added to the remaining cells for 3 additional days to induce sgRNA editing before sequencing. Genomic DNA was isolated from cell pellets using a genomic DNA isolation kit (Blood & Cell Culture Midi kit, QIAGEN). After gDNA isolation, sgRNAs were amplified and barcoded by PCR as in ref. ^67^, to amplify the DNA fragment containing sgRNA sequences. PCR products were sequenced on a HiSeq 4000 instrument (Illumina) at 50-bp reads to a depth of 30 million reads per sample. Reads were preprocessed by removing adaptors using Cutadapt (v.4.1)^68^ with parameters ‘--e 0.2 --a GTTTTAGAGCTAGAAATAGCAAGTTAAAATA --m 18’. Next, sequences were trimmed to length 19 to match that of the probes. An artificial genome was created for alignment with Bowtie (v.0.12.9)^69^ using the probes’ sequences and the function ‘bowtie-build’ with default parameters. Finally, reads were aligned to this genome with parameters --S --t --p 20 --n 1 --l 19. Read counts were imported into R (ref. ^70^) by reading the sam files and counting the number of occurrences of each probe. The resulting count matrix was normalized using the rlog function from the DESeq2 v.1.34.0 R package^71^. A linear model with random and fixed effects was fitted to the normalized probe data for each gene. The condition was used as a fixed covariable while the guides were included as random effects whenever there was more than one. The model was fit with the function lmer from the lme4 package^71^ or with the native R lm function if there was only one probe for a given gene. Contrast coefficients and *P* values were computed using the glht function from the multcomp package^72^ without any *P*-value adjustment. Gene Ontology gene-set collections were downloaded from the Gene Ontology knowledgebase^73^. Genes quantified in the microarray study were annotated according to the Broad Hallmark^74^. Functional enrichment analyses were performed using a modification of ROAST^75^, a rotation-based approach implemented in the Limma R package^76^, which is especially suitable for small experiments. Such modifications were implemented to accommodate the proposed statistical restandardization^77^ in the ROAST algorithm, which enables its use for competitive testing^78^. The MaxMean statistic was used for testing gene-set enrichment of the different gene collections^77^. For each gene, the most variable guide within each gene was used in these analyses (median absolute deviation). The results of these analyses were adjusted by multiple comparisons using the Benjamini–Hochberg False Discovery Rate method^79^. All these was performed using the functions of the roastgsa R package^80^.

### siRNA treatment

siRNA for *TFEB* was purchased from siTOOLs. siRNAs (non-targeting (NT) and *TFEB*) were used at a final concentration of 3 nM. Both proliferating and diapause-like SK-Mel-147 were transfected with siRNAs for 5 days before performing viability assays. Lipofectamine reagent RNAiMAX (13778075) was used at 2 μl per ml to perform the transfection. Cell viability was measured using CellTiter-Glo Luminescent cell viability assay (Promega). Raw data were acquired by measuring luminescence in a VICTOR Multilabel Plate Reader (Pelkin Elmer). For the diapause exit experiment, SK-Mel-147 cells were either proliferating or treated with INK128 for 7 days and then transfected with siNT or siTFEB. We tested four different conditions, first proliferating cells, INK128-treated cells, cells washed out of INK128 at day 0 of siRNA transfection and cells washed out of INK128 3 days after siRNA infection. Images were taken 6 days after siRNA transfection using a Nikon Eclipse TS2 brightfield microscope (RRID: SCR_025716).

### Statistics and reproducibility

The statistical tests performed in this study are indicated in figure legends and method details. Data are represented as the mean ± s.d. if not stated otherwise in the figure legends. Number of replicates and animals for each experiment are enclosed in their respective figure legends and or method details. No statistical methods were used to predetermine sample sizes, but our sample sizes are similar to those reported in previous publications^16^. Data collection and analysis were performed blind to the conditions of the experiments.

### Reporting summary

Further information on research design is available in the Nature Portfolio Reporting Summary linked to this article.

## Supplementary information

Reporting Summary

Supplementary Tables 1–4 Table 1. Hallmarks of cellular senescence. Table 2. Experimental data and statistics. Table 3. Overview of gene ontology biological processes (GOTERM_BP_DIRECT). Table 4. *C. elegans* SenMayo gene-set orthologs.

## Source data

Source Data Fig. 1 Source data for graphs plotted.

Source Data Fig. 2 Source data for graphs plotted.

Source Data Fig. 3 Source data for graphs plotted.

Source Data Fig. 4 Source data for graphs plotted.

Source Data Fig. 5 Source data for graphs plotted.

Source Data Extended Data Fig. 1 Source data for graphs plotted.

Source Data Extended Data Fig. 2 Source data for graphs plotted.

Source Data Extended Data Fig. 3 Source data for graphs plotted.

Source Data Extended Data Fig. 4 Source data for graphs plotted.

Source Data Extended Data Fig. 5 Source data for graphs plotted.

Source Data Extended Data Fig. 6 Source data for graphs plotted.

Unprocessed blots uncropped western blots.

## Data Availability

All data generated in this study are available in the main text or the Supplementary Information. *C. elegans* RNA-seq data accession code: GSE291659; WBcel235.80 reference genome *C. elegans* RNA-seq; WS220/ce10 *C. elegans* assembly was used as reference genome mutagenesis; (Nfu_20140520) reference genome for killifish RNA-seq, RNA-seq data accession code: GSE296348; Killifish diapause transcriptomic data were obtained from ref. ^53^ and ref. ^54^.

## Extended data

are available for this paper at 10.1038/s43587-025-00911-4.
