# Supplementary material for: A TFEB–TGFβ axis systemically regulates diapause, stem cell resilience and protects against a senescence-like state
Source: Nat Aging. 2025 Jun 30;5(7):1340–57. doi: 10.1038/s43587-025-00911-4 (PMC12270908; doi:10.1038/s43587-025-00911-4)
Supplement: Supplementary file 1 — Reporting Summary [file 43587_2025_911_MOESM1_ESM.pdf]

nature portfolio

Corresponding author(s): Adam Antebi
Last updated by author(s): May 7, 2025

Reporting Summary

Nature Portfolio wishes to improve the reproducibility of the work that we publish. This form provides structure for consistency and transparency in reporting. For further information on Nature Portfolio policies, see our Editorial Policies and the Editorial Policy Checklist.

Statistics

For all statistical analyses, confirm that the following items are present in the figure legend, table legend, main text, or Methods section.

n/a	Confirmed	
☐	☒	The exact sample size (*n*) for each experimental group/condition, given as a discrete number and unit of measurement
☐	☒	A statement on whether measurements were taken from distinct samples or whether the same sample was measured repeatedly
☐	☒	The statistical test(s) used AND whether they are one- or two-sided<br>*Only common tests should be described solely by name; describe more complex techniques in the Methods section.*
☒	☐	A description of all covariates tested
☐	☒	A description of any assumptions or corrections, such as tests of normality and adjustment for multiple comparisons
☐	☒	A full description of the statistical parameters including central tendency (e.g. means) or other basic estimates (e.g. regression coefficient) AND variation (e.g. standard deviation) or associated estimates of uncertainty (e.g. confidence intervals)
☐	☒	For null hypothesis testing, the test statistic (e.g. *F*, *t*, *r*) with confidence intervals, effect sizes, degrees of freedom and *P* value noted<br>*Give P values as exact values whenever suitable.*
☒	☐	For Bayesian analysis, information on the choice of priors and Markov chain Monte Carlo settings
☒	☐	For hierarchical and complex designs, identification of the appropriate level for tests and full reporting of outcomes
☒	☐	Estimates of effect sizes (e.g. Cohen's *d*, Pearson's *r*), indicating how they were calculated

Our web collection on statistics for biologists contains articles on many of the points above.

Software and code

Policy information about availability of computer code

Data collection	HiSeq 4000 & 2500 (Illumina), Cutadapt v4.1, Bowtie v0.12.9, Flexbar v2.5, HISAT2 v2.0.4, Kallisto v0.45.0, DESeq2 v1.24.0 & v1.34.0, cuffmerge v 2.2.1
Data analysis	ImageJ2 v2.14.0/1.54h; Mitochondria analyzer V2.3.1 ImageJ2 plug-in; R packages: lme4 v1.1-36, multcomp v1.4-28, Limma v4.4, ROAST v4.4, roastgsa v4.4;GO gene sets were created from Gene ontology knowledgebase; Graph Pad Prism v10.3.1 or Flaski v3.17.12; Flexbar version 2.5; Cuffquant version 2.2.1 and Cuffdiff version 2.2.1; Galaxy v24.1 software; MiModD v0.1.9; BiT age v2; Bowtie v0.12.9, Zen2.3, Flow Pilot, LAS X, Imaris 10.0.0, Adobe Illustrator 26.0.3, Excel 16.94, StringTie v1.04, Cufflinks v2.2.1

For manuscripts utilizing custom algorithms or software that are central to the research but not yet described in published literature, software must be made available to editors and reviewers. We strongly encourage code deposition in a community repository (e.g. GitHub). See the Nature Portfolio guidelines for submitting code & software for further information.

Data

Policy information about availability of data

All manuscripts must include a data availability statement. This statement should provide the following information, where applicable:

- Accession codes, unique identifiers, or web links for publicly available datasets
- A description of any restrictions on data availability
- For clinical datasets or third party data, please ensure that the statement adheres to our policy

All data generated in this study are available in the main text or the supplementary materials. C. elegans RNA-seq data accession code: GSE291659; WBcel235.80 reference genome C. elegans RNA seq; WS220/ce10 C. elegans assembly was used as reference genome mutagenesis; (Nfu_20140520) reference genome for killifish RNA seq, RNA-seq data accession code: GSE296348; Killifish diapause transcriptomic data was obtained from "K. Reichwald et al., Insights into Sex Chromosome Evolution and Aging from the Genome of a Short-Lived Fish. Cell 163, 1527-1538 (2015)" and "C. K. Hu et al., Vertebrate diapause preserves organisms long term through Polycomb complex members. Science 367, 870-874 (2020)".

Research involving human participants, their data, or biological material

Policy information about studies with human participants or human data. See also policy information about sex, gender (identity/presentation), and sexual orientation and race, ethnicity and racism.

Reporting on sex and gender	N/A
Reporting on race, ethnicity, or other socially relevant groupings	N/A
Population characteristics	N/A
Recruitment	N/A
Ethics oversight	N/A

Note that full information on the approval of the study protocol must also be provided in the manuscript.

Field-specific reporting

Please select the one below that is the best fit for your research. If you are not sure, read the appropriate sections before making your selection.

☒ Life sciences ☐ Behavioural & social sciences ☐ Ecological, evolutionary & environmental sciences

For a reference copy of the document with all sections, see nature.com/documents/nr-reporting-summary-flat.pdf

Life sciences study design

All studies must disclose on these points even when the disclosure is negative.

Sample size	Sample size were chosen in accordance with previously published work (B. Gerisch et al., HLH-30/TFEB Is a Master Regulator of Reproductive Quiescence. Dev Cell 53, 316-329 e315 (2020).)
Data exclusions	In the C. elegans RNAseq data, two outliers were excluded from the study (hlh-30 Recovery replicate 4 and daf-1 ARD replicate 3)
Replication	All findings in the present study were reproducible and performed in minimum two independent biological replicates
Randomization	Killifish were assigned randomly into starvation and control groups. Worms were randomly chosen for microscopic analysis.
Blinding	All experiments were performed blinded during, group allocation, the experimental procedure and data analysis.

Reporting for specific materials, systems and methods

We require information from authors about some types of materials, experimental systems and methods used in many studies. Here, indicate whether each material, system or method listed is relevant to your study. If you are not sure if a list item applies to your research, read the appropriate section before selecting a response.

Materials & experimental systems

n/a	Involved in the study	
☐	☒	Antibodies
☐	☒	Eukaryotic cell lines
☒	☐	Palaeontology and archaeology
☐	☒	Animals and other organisms
☒	☐	Clinical data
☒	☐	Dual use research of concern
☒	☐	Plants

Methods

n/a	Involved in the study	
☒	☐	ChIP-seq
☒	☐	Flow cytometry
☒	☐	MRI-based neuroimaging

Antibodies

Antibodies used	Phospho-Histone H3 (Ser10), 6G3 Mouse mAb, Cell Signaling, 9706; ANTI-FLAG, M2, Sigma-Aldrich, F3165; Alexa Fluor 488, Invitrogen, A-11008; Alexa Fluor 594, Invitrogen, A-11005; Anti RAD-51, a custom made from Smolikove lab (U. of Iowa); Goat anti-Rabbit IgG (H+L), HRP, Invitrogen, G-21234; Goat anti-Mouse IgG (H+L), HRP, Invitrogen, G-21040; Anti-α-tubulin, DM1A, Sigma-Aldrich, T9026
Validation	Phospho-H3 and Anti-Flag validated by manufacturer for use in C. elegans; Secondary antibodies (Alexa Fluor 488, Alexa Fluor 594, Goat anit-Rabbit and Goat anti-Mouse) have been validated to bind there target protein by manufacturers. For Anti RAD-51 see Tara Hick et al., 2022; Alexa Fluor 488 and 594 were validated for use in C. elegans by multiple authors (Jer-Yuan Hsu et al., 2000, Seidel et al., 2015); Anti-α-tubulin has been validated to bind its target protein by the manufacturers

Eukaryotic cell lines

Policy information about cell lines and Sex and Gender in Research

Cell line source(s)	SK-MEL-147, Source ATCC, Melanoma cell line; mESC strain: KH2 ESC, provided by Oscar Fernandez-Capetillo (CNIO, Madrid)
Authentication	All the cell lines have been authenticated through a dedicated service provided by the Institute for Biomedical Research in Madrid
Mycoplasma contamination	negative (monthly tested)
Commonly misidentified lines (See ICLAC register)	The study does not include any commonly misidentified lines

Animals and other research organisms

Policy information about studies involving animals; ARRIVE guidelines recommended for reporting animal research, and Sex and Gender in Research

Laboratory animals	African turquoise killifish Nothobranchius furzeri, Strain: GRZ-AD, 6-8 weeks & 18-20 weeks; Caenorhabditis elegans:N2, AA3658, DR40, CB1372, JT9609, PR678, AA4401, AA5311, PHX2718, AA5417, CB1376, AA4397, AA60, AA5029, AA5030, AA5033, AA5325, AA5450, AA5339, AA5252, AA5289, AA5034, AA5036, AA5035, AA5466, AA5037, AA5038, DR1767, AA5553, DR2021, AA4369, GC1038, AA5336, AA5338, PHX809, CB4856, AA4398, WU1770, AA5415, QU190, AA4371, SJZ106, AA5464, DR466, AA3751, HZ1691,HZ1688,HZ1691,HZ1687,HZ1683,QU190,HZ931,RB2372,AA4371,HZ903,PHX773,RB807,VC1003,RB938state: ARD or ad libitum young adult
Wild animals	The study does not include any wild animals
Reporting on sex	Killifish sex: males; C. elegans: hermaphordite
Field-collected samples	The study does not include any field-collected samples
Ethics oversight	Landesamt für Natur, Umwelt und Verbraucherschutz Nordrhein- Westfalen": 81-02.04.2019.A055

Note that full information on the approval of the study protocol must also be provided in the manuscript.

Plants

Seed stocks	N/A
Novel plant genotypes	N/A
Authentication	N/A
