# Supplementary figures and images for: A TFEB–TGFβ axis systemically regulates diapause, stem cell resilience and protects against a senescence-like state
Source: Nat Aging. 2025 Jun 30;5(7):1340–57. doi: 10.1038/s43587-025-00911-4 (PMC12270908; doi:10.1038/s43587-025-00911-4)

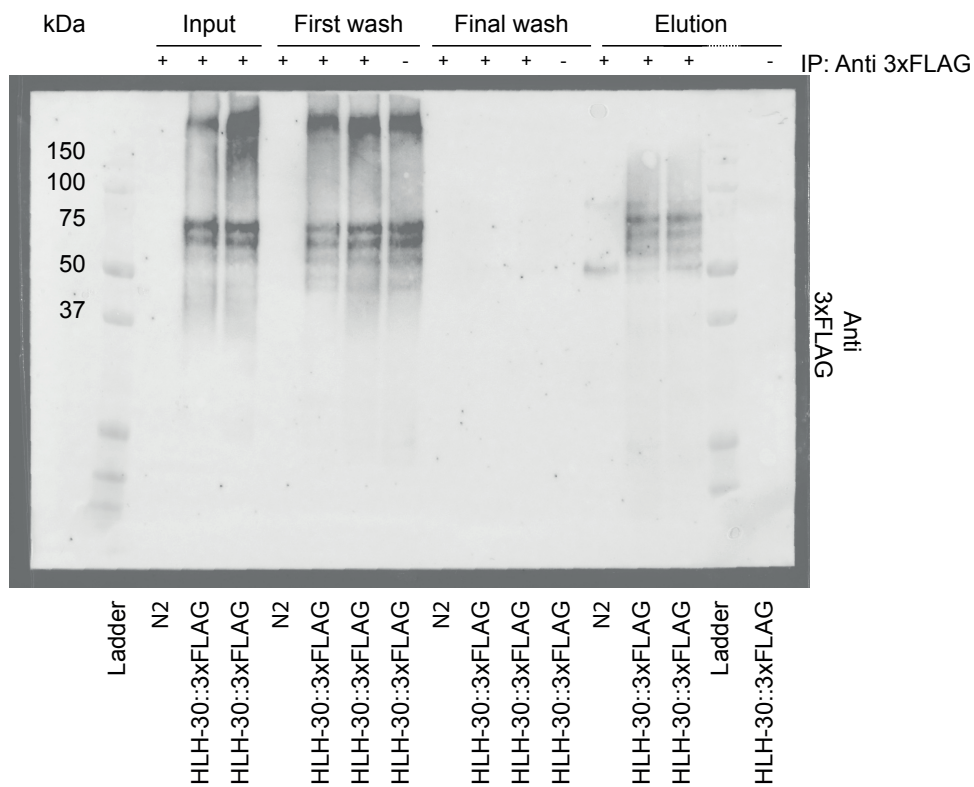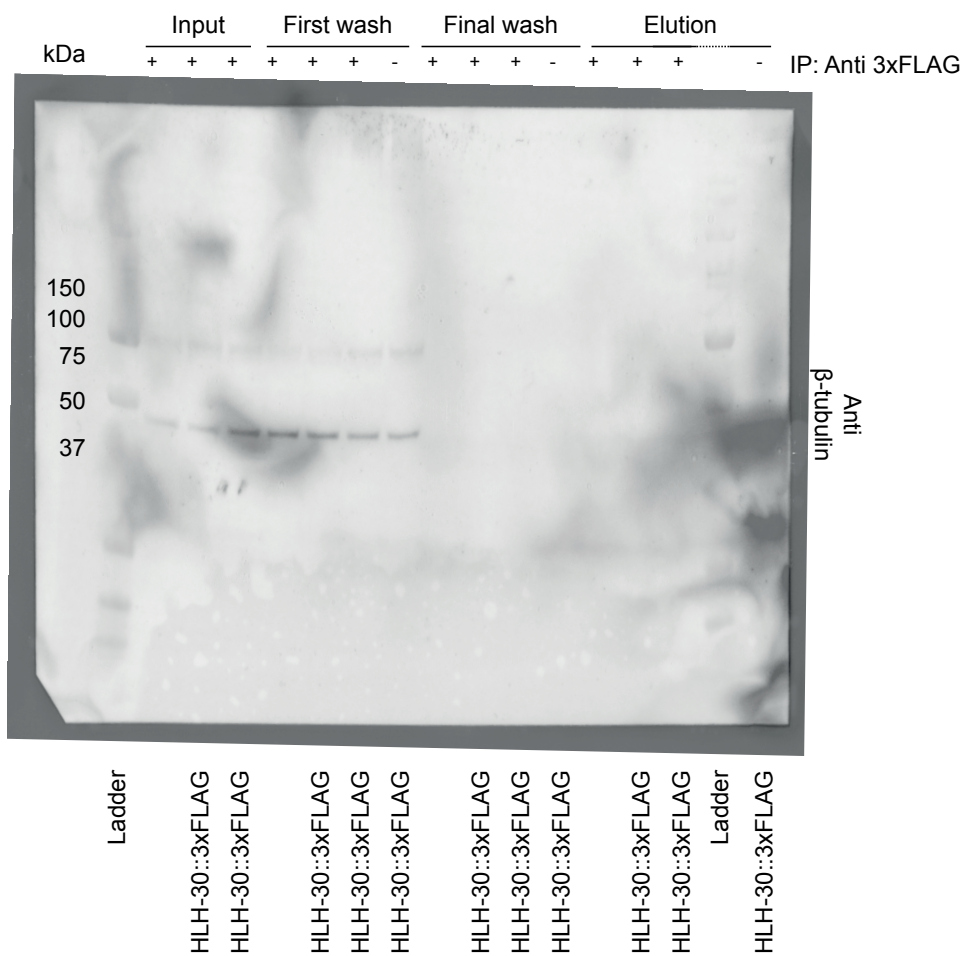

Supplement: Supplementary file 14 — uncropped western blots. [file 43587_2025_911_MOESM14_ESM.pdf]
